# Supplementary material for: Structure of the proteolytic enzyme PAPP-A with the endogenous inhibitor stanniocalcin-2 reveals its inhibitory mechanism
Source: Nat Commun. 2022 Oct 18;13:6084. doi: 10.1038/s41467-022-33698-8 (PMC9579167; doi:10.1038/s41467-022-33698-8)
Supplement: Supplementary file 1 — Supplementary Information [file 41467_2022_33698_MOESM1_ESM.pdf]

SUPPLEMENTARY INFORMATION

Structure of the proteolytic enzyme PAPP-A with the endogenous inhibitor stanniocalcin-2 reveals its inhibitory mechanism

Sara Dam Kobberø[1,6], Michael Gajhede[2,6], Osman Asghar Mirza[2], Søren Kløverpris[1,3], Troels Rønn Kjær[1,4], Jakob Hauge Mikkelsen[1,4], Thomas Boesen[5] & Claus Oxvig[1*]

[1]Department of Molecular Biology and Genetics, Aarhus University, DK-8000 Aarhus C, Denmark; [2]Department of Drug Design and Pharmacology, University of Copenhagen, DK-2100 Copenhagen Ø, Denmark; [3]Present address: Agilent Technologies; DK-2600 Glostrup, Denmark; [4]Present address: Department of Biomedicine, Aarhus University, DK-8000 Aarhus C, Denmark; [5]Interdisciplinary Nanoscience Center, Aarhus University, DK-8000 Aarhus C. [6]These authors contributed equally: Sara Dam Kobberø and Michael Gajhede. [*]Email: co@mbg.au.dk

All Supplementary Information is contained in this file, except Supplementary Data 1 and Supplementary Movie 1.

a (Mass photometry of PAPP-A and PAPP-A·STC2)

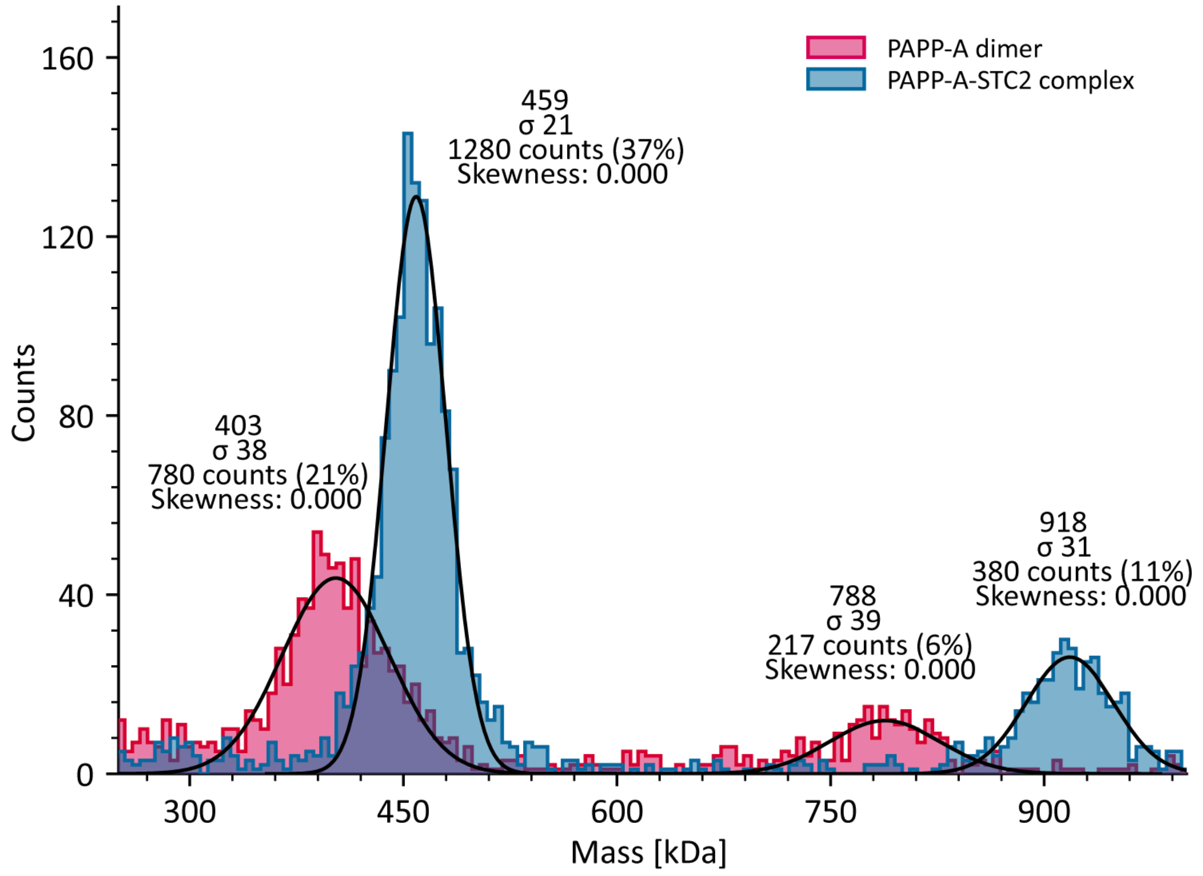

b (N- and C-terminal peptides identified in PAPP-A·STC2 tryptic digest)

PAPP-A (UniProt Q13219): N-terminal 180 residues (of preproPAPP-A):

```
        10         20         30         40         50         60
MRLWSWVLHL GLLSAALGCG LAERPRRARR DPRAGRPPRP AAGPATCATR AARGRRASPP

        70         80         90        100        110        120
PPPPPGGAWE AVRVPRRRQQ REARGATEEP SPPSRALYFS GRGEQLRLRA DLELPRDAFT

       130        140        150        160        170        180
LQVWLRAEGG QRSPAVITGL YDKCSYISRD RGWVVGIHTI SDQDNKDPRY FFSLKTDRAR
```

PAPP-A (UniProt Q13219): C-terminal 127 residues:

```
      1510       1520       1530       1540       1550       1560
ECATSCLDHN SESIILPMNV TVRDIPHWLN PTRVERVVCT AGLKWYPHPA LIHCVKGCEP

      1570       1580       1590       1600       1610       1620
FMGDNYCDAI NNRAFCNYDG GDCCTSTVKT KKVTPFPMSC DLQGDCACRD PQAQEHSRKD

   1627
LRGYSHG
```

STC2 (UniProt O76061): Full sequence:

```
        10         20         30         40         50         60
MCAERLGQFM TLALVLATFD PARGTDATNP PEGPQDRSSQ QKGRLSLQNT AEIQHCLVNA

        70         80         90        100        110        120
GDVGCGVFEC FENNSCEIRG LHGICMTFLH NAGKFDAQGK SFIKDALKCK AHALRHRFGC

       130        140        150        160        170        180
ISRKCPAIRE MVSQLQRECY LKHDLCAAAQ ENTRVIVEMI HFKDLLLHEP YVDLVNLLLT

       190        200        210        220        230        240
CGEEVKEAIT HSVQVQCEQN WGSLCSILSF CTSAIQKPPT APPERQPQVD RTKLSRAHHG

       250        260        270        280        290        300
EAGHHLPEPS SRETGRGAKG ERGSKSHPNA HARGRVGGLG AQGPSGSSEW EDEQSEYSDI

RR
```

Supplementary Fig. 1 Analysis of the recombinant PAPP-A·STC2 complex. a Molecular mass determination of uncomplexed PAPP-A dimer (red bars) and PAPP-A covalently bound to STC2 (blue bars). The data shows that the PAPP-A dimer (estimated molecular mass of 403 kDa) upon binding to STC2 increases its molecular mass to an estimated value of 459 kDa. This is in agreement with the binding of a single STC2 dimer (the calculated peptide mass of an STC2 dimer of residues G24-R302 is 61.4 kDa), thus reflecting that the PAPP-A·STC2 complex has a 2:2 stoichiometry. (The peaks at 788 kDa and 918 kDa represent transient dimers of the PAPP-A dimer and the PAPP-A·STC2 2:2 complex, respectively). The experiment was repeated three times with similar results. **b** Key peptides identified in a trypic digest (Supplementary Table 3) of purified PAPP-A·STC2 by mass spectrometry. Identified peptides from the N- or C-terminal ends of the subunits are highlighted (grey). Native mature PAPP-A starts at E81. The prepro part of native PAPP-A, and the signal peptide of STC2 is underlined. The N-terminal peptide of PAPP-A carries an additional alanine residue before E81 as determined by previous N-terminal sequence analysis of the recombinant protein[1]. The first four PAPP-A residues (AE82AR), escaped mass spectrometric detection. The very C-terminal peptides of PAPP-A (D1620-G1627) and STC2 (W290-R302) were not identified. Based on this and on the presence of intact bands of the expected molecular weights following SDS-PAGE (Fig. 2a), we conclude that except for the C-terminal eight residues of the PAPP-A subunit and 13 residues of the STC2 subunit, no peptide mass of the PAPP-A·STC2 complex is missing.

a (Representative micrographs from Dataset I and Dataset II)

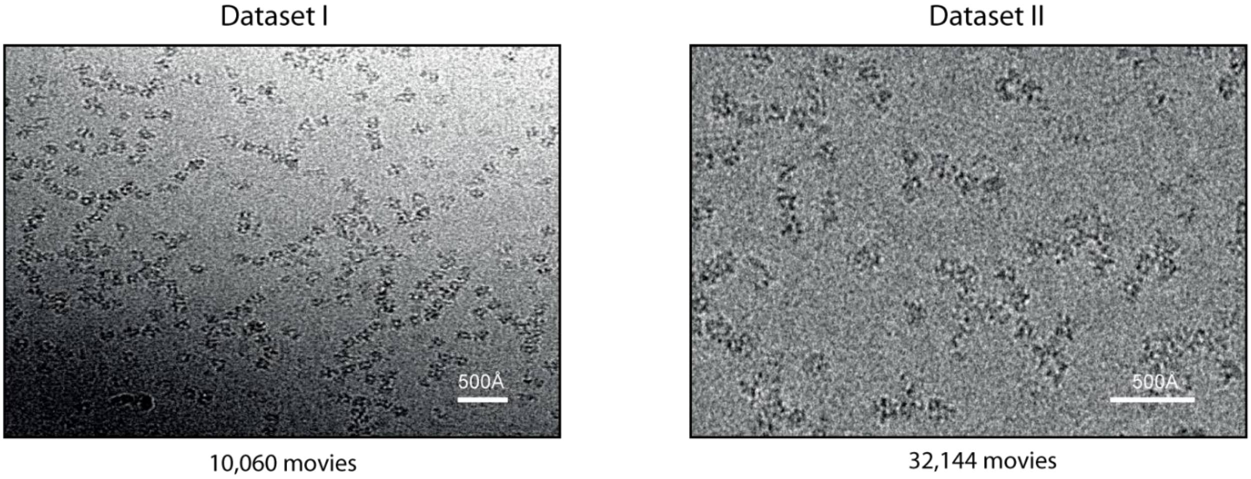

b (Representative 2D classes from combined Dataset I and Dataset II)

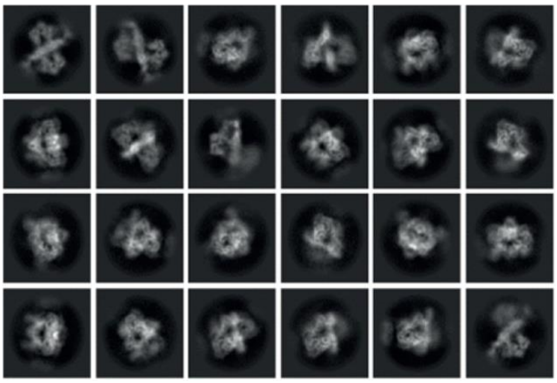

c (Workflow for MAP1 (3.06 Å))

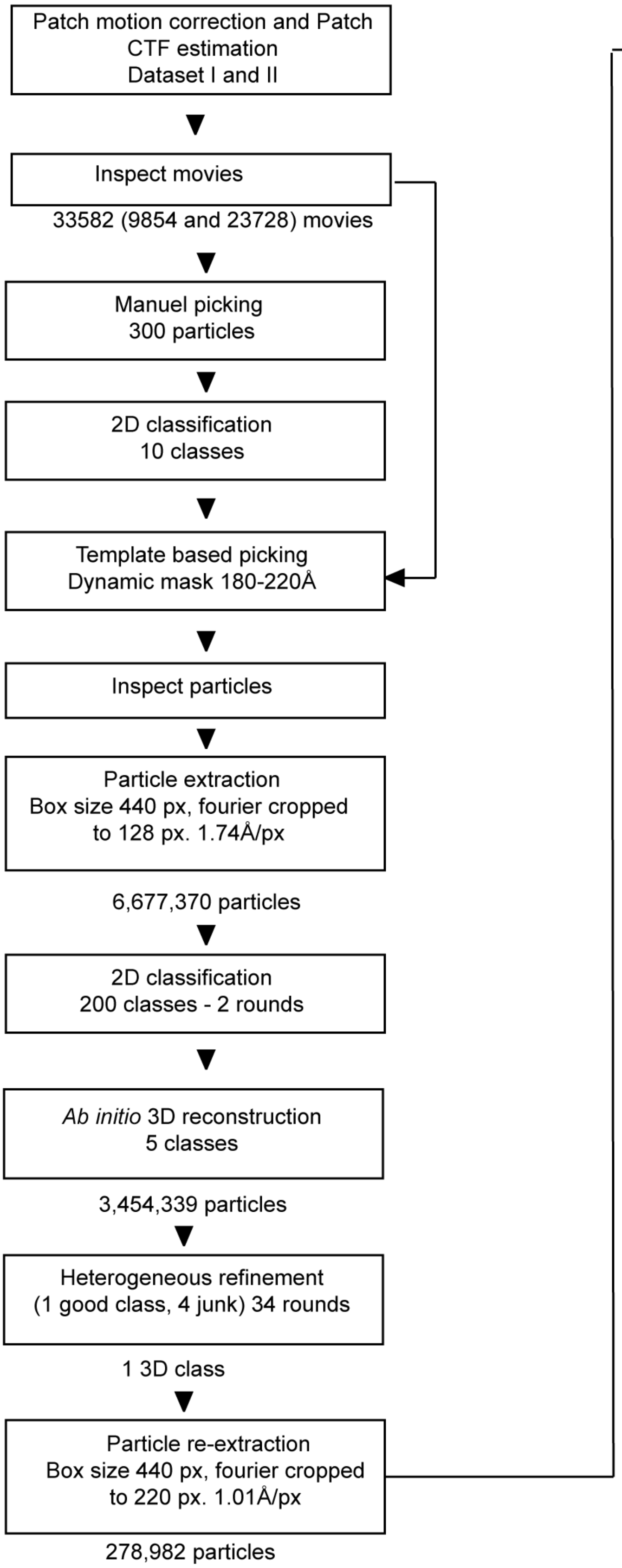

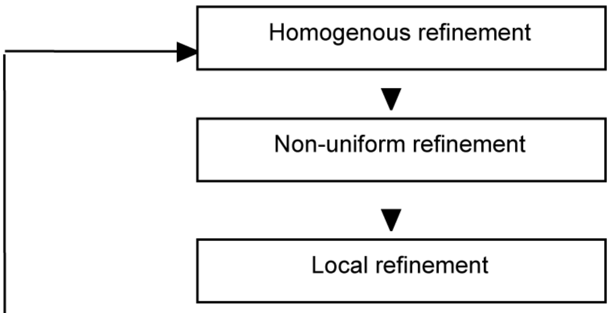

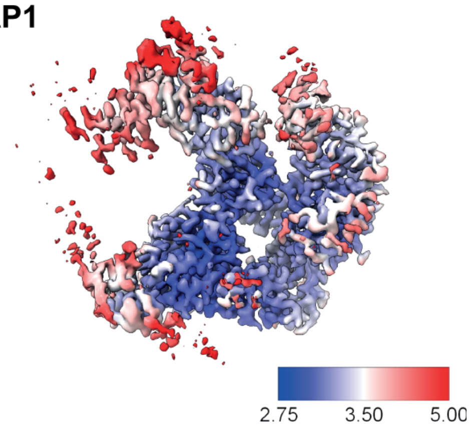

d (MAP1: Gold standard Fourier shell correlation curve and viewing direction distribution)

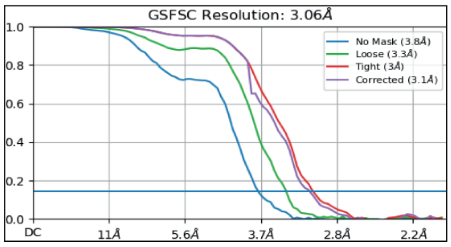

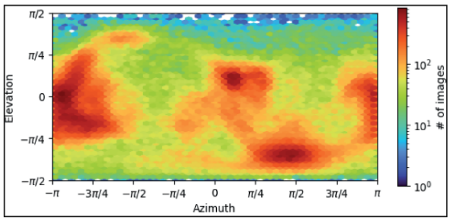

e (Workflow for MAP2 (4.05Å) and MAP3 (5.02Å))

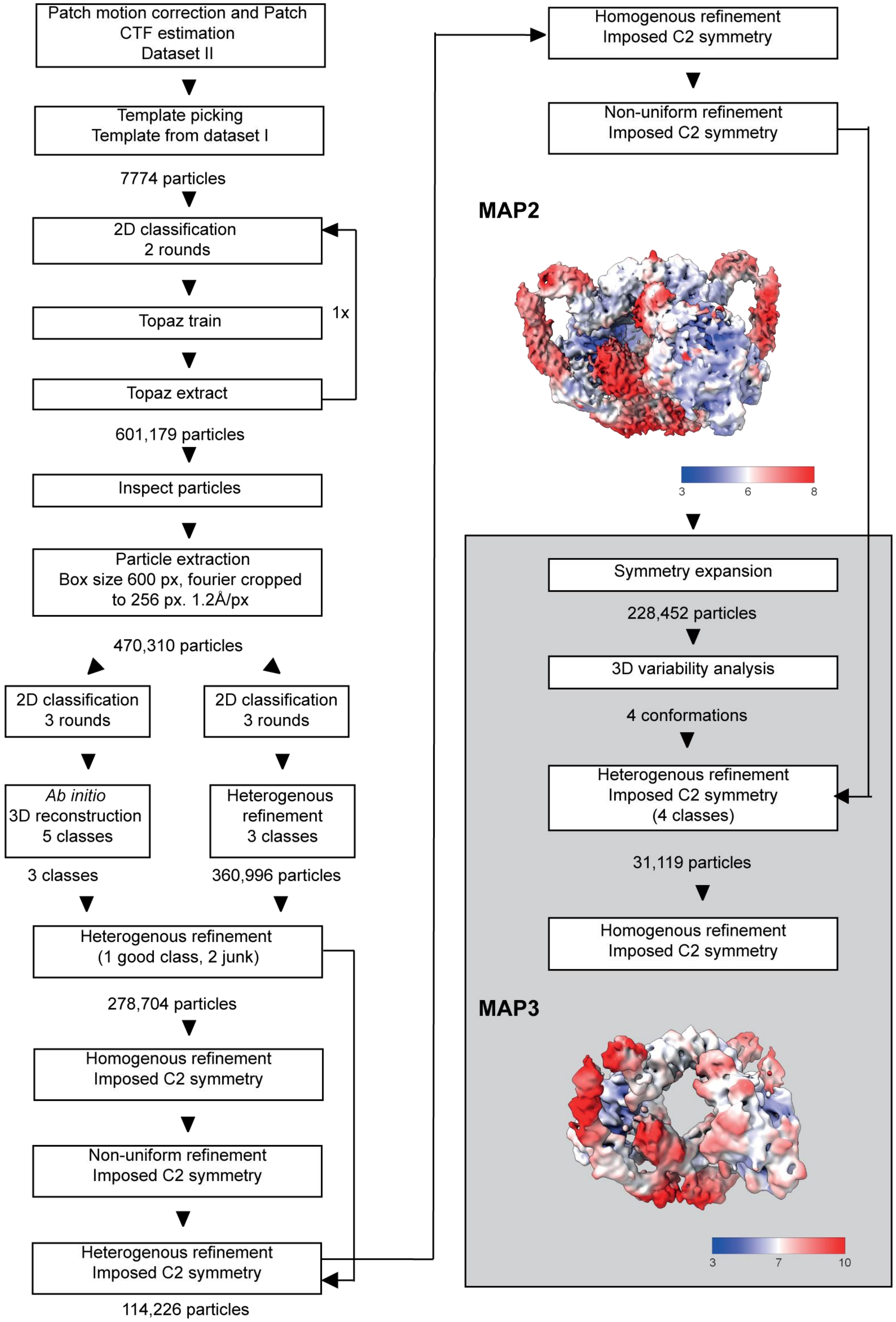

f (MAP2 and MAP3: Gold standard Fourier shell correlation curve and viewing direction distribution)

MAP2

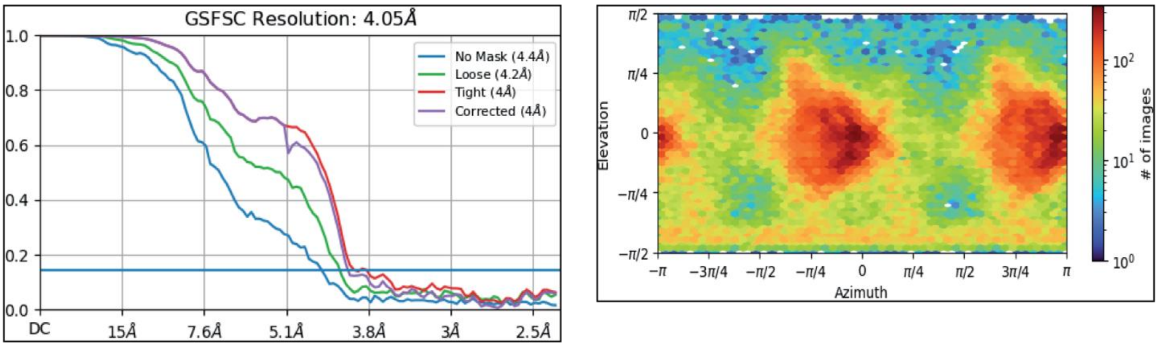

MAP3

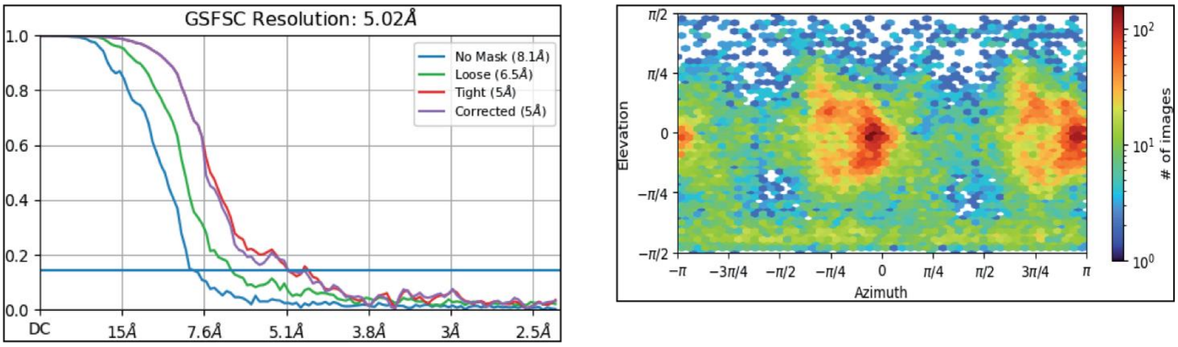

Supplementary Fig. 2 Workflow from cryo-EM processing in cryoSPARC v3. a Representative micrographs from Dataset I and Dataset II. **b** Representative 2D classes from combined Dataset I and Dataset II. **c** Workflow for MAP1 (3.06 Å). **d** MAP1: Gold standard Fourier shell correlation curve and viewing direction distribution. **e** Workflow for MAP2 (4.05Å) and MAP3 (5.02Å). **f** MAP2 and MAP3: Gold standard Fourier shell correlation curve and viewing direction distribution.

a (MAP1)

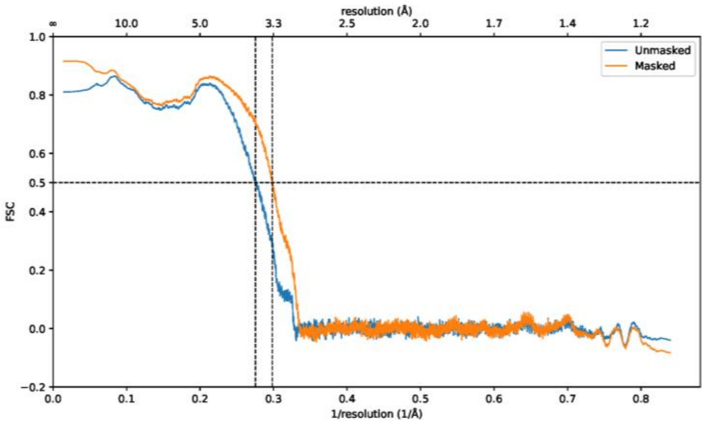

b (Composite map)

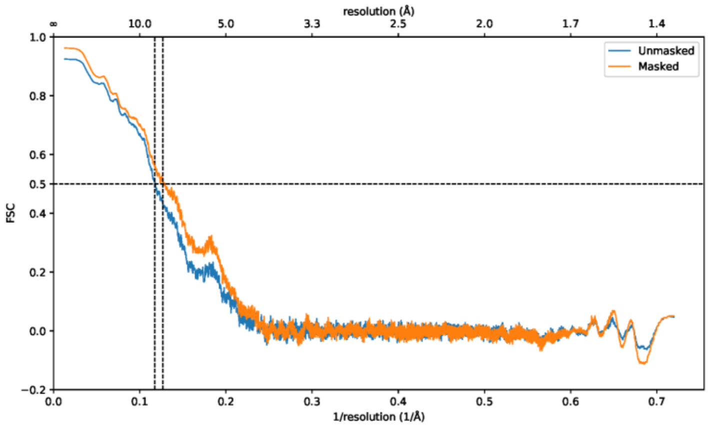

c (MAP2)

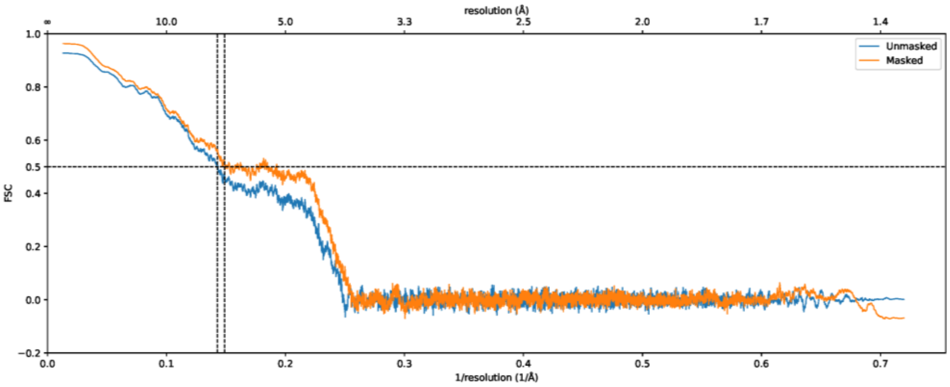

d (MAP3)

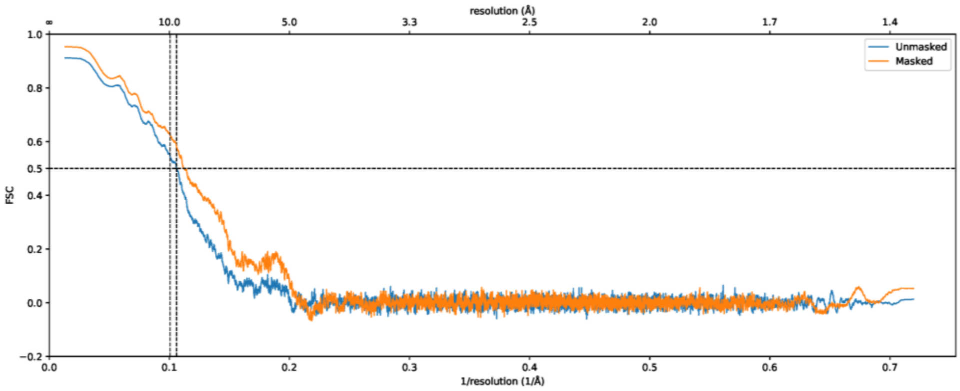

Supplementary Fig. 3 Estimation of map resolution from calculated Fourier shell correlations (FSC) between density maps and atomic models. **a** MAP1 compared to the partial dimer atomic model (PDB ID: 8A7D). Resolution at FSC 0.143 is 3.04 Å. **b** Composite map (combined MAP1, MAP2, and MAP3) compared to final dimer model (PDB ID: 8A7E). Resolution at FSC 0.143 is 4.95 Å. **c** MAP2 compared to final dimer model. Resolution at FSC 0.143 is 4.12 Å. **d** MAP3 compared to final dimer model. Resolution at FSC 0.143 is 6.42 Å.

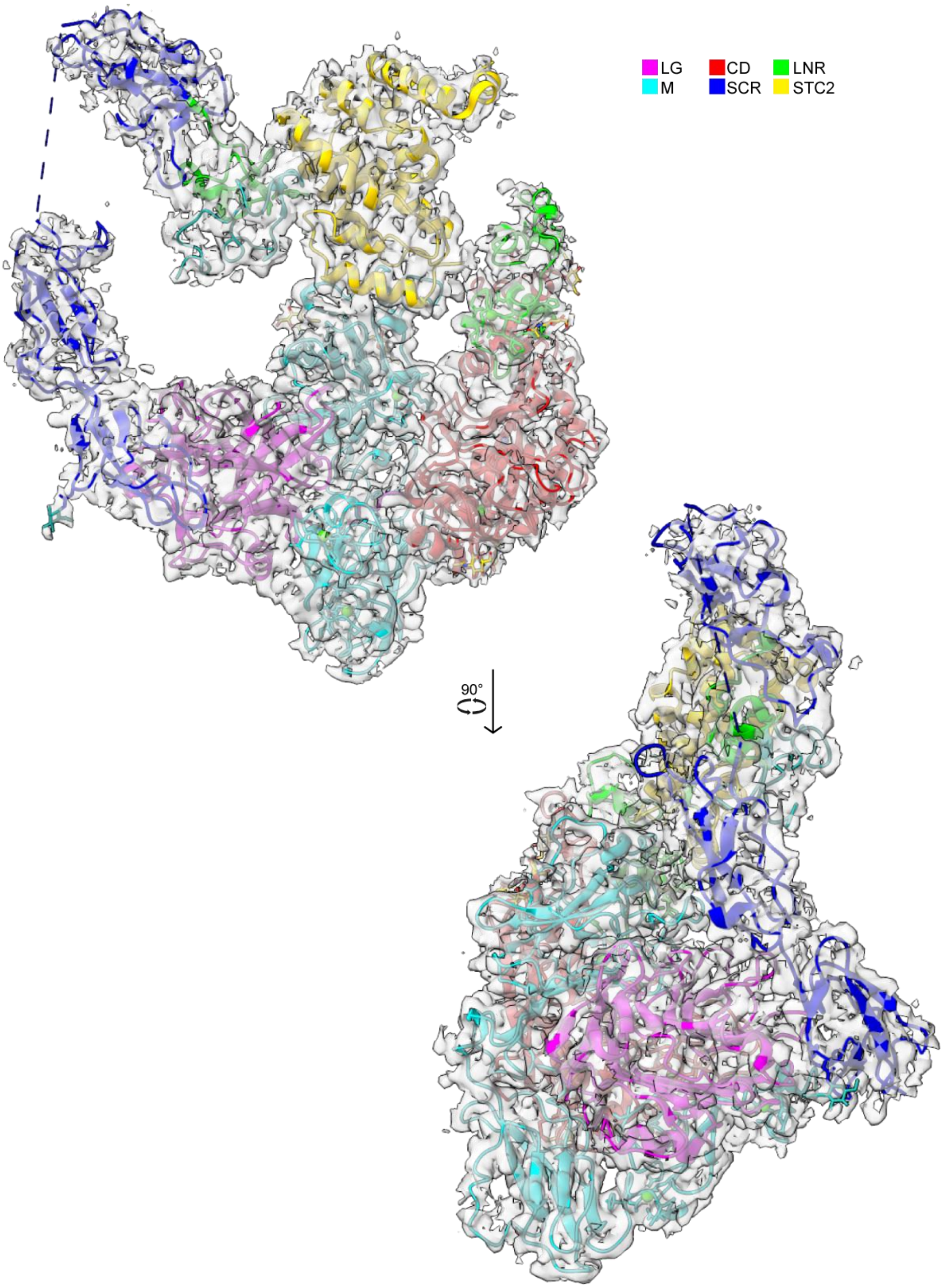

Supplementary Fig. 4 Fit between the partial dimer model (PDB ID: 8A7D) and MAP1. A cartoon representation of the model is shown with MAP1 at a contour level of 2.7 σ. MAP1 has a resolution of 3.04 Å according to FSC at 0.143.

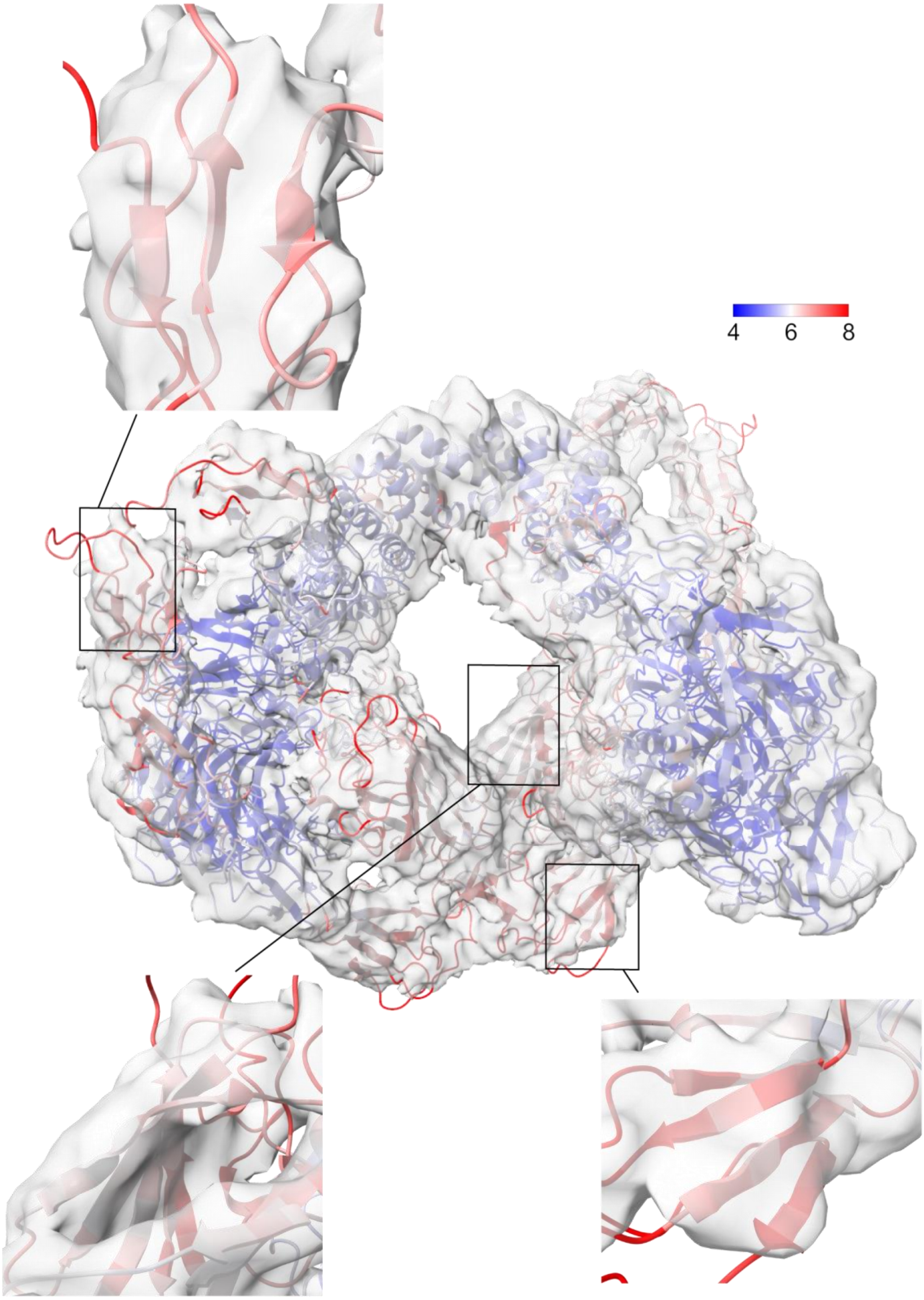

Supplementary Fig. 5 Quality of density map in relation to FSC-based calculated resolution. The full PAPP-A·STC2 complex atomic model with estimation of resolution indicated (color bar indicates Å values) fitted into the composite density map that merges MAP1, MAP2 and MAP3. Resolution value is interpolated to each atom based on the local resolution of the composite map. The map (composite map) was contoured at 5 σ. Inserts zooms at selected lower resolution areas and demonstrates that, e.g., the two sheets of the M5 beta-sandwich are clearly resolved. The map in the zoomed regions is contoured at different levels (MAP3 or composite map, 4-8 σ) to highlight secondary structural elements.

a

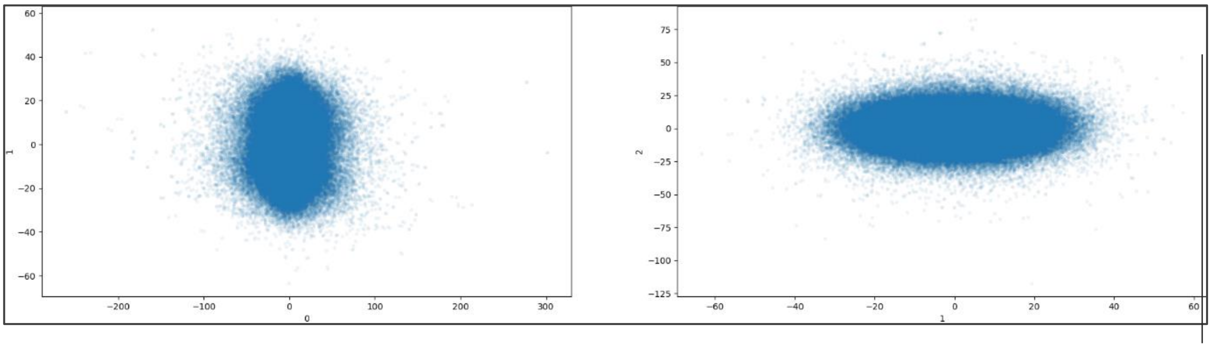

b

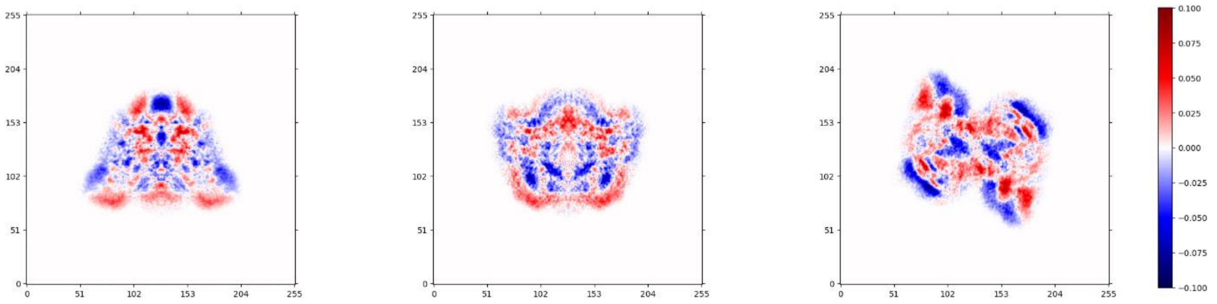

Supplementary Fig. 6 3D variability analysis of MAP2. A total of three components were calculated. **a** Reaction coordinates of component 0 vs component 1 and of component 0 vs. component 1. No clustering is observed, suggesting non-discrete or continuous flexibility. **b** Orthogonal slices of component 2. Positive (red) and negative (blue) values correspond to density to be added and subtracted from the consensus density to model the heterogeneity in the particle set. Large differences can be observed.

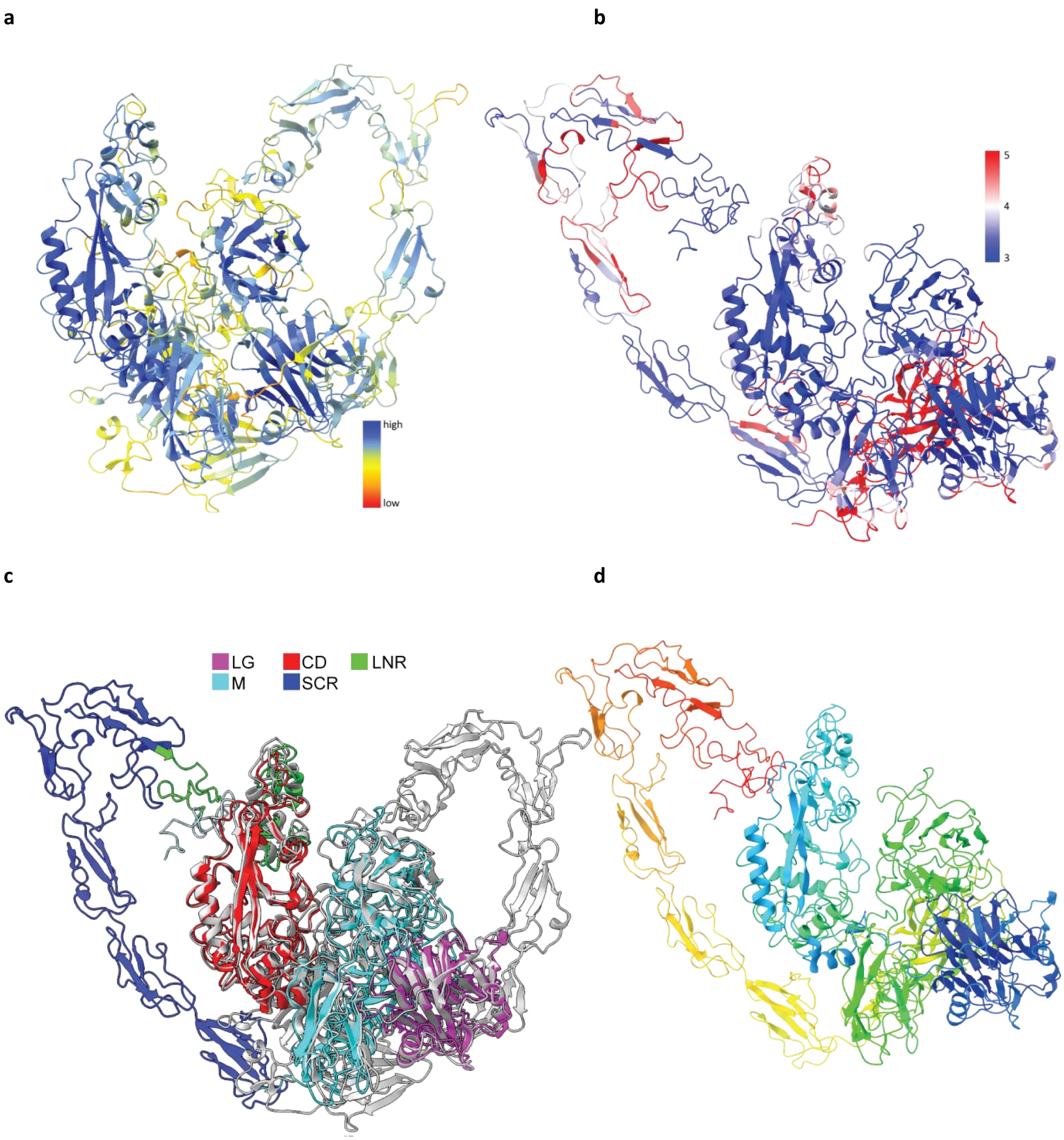
a
b
c
d
high
low
5
4
3
LG
CD
LNR
M
SCR

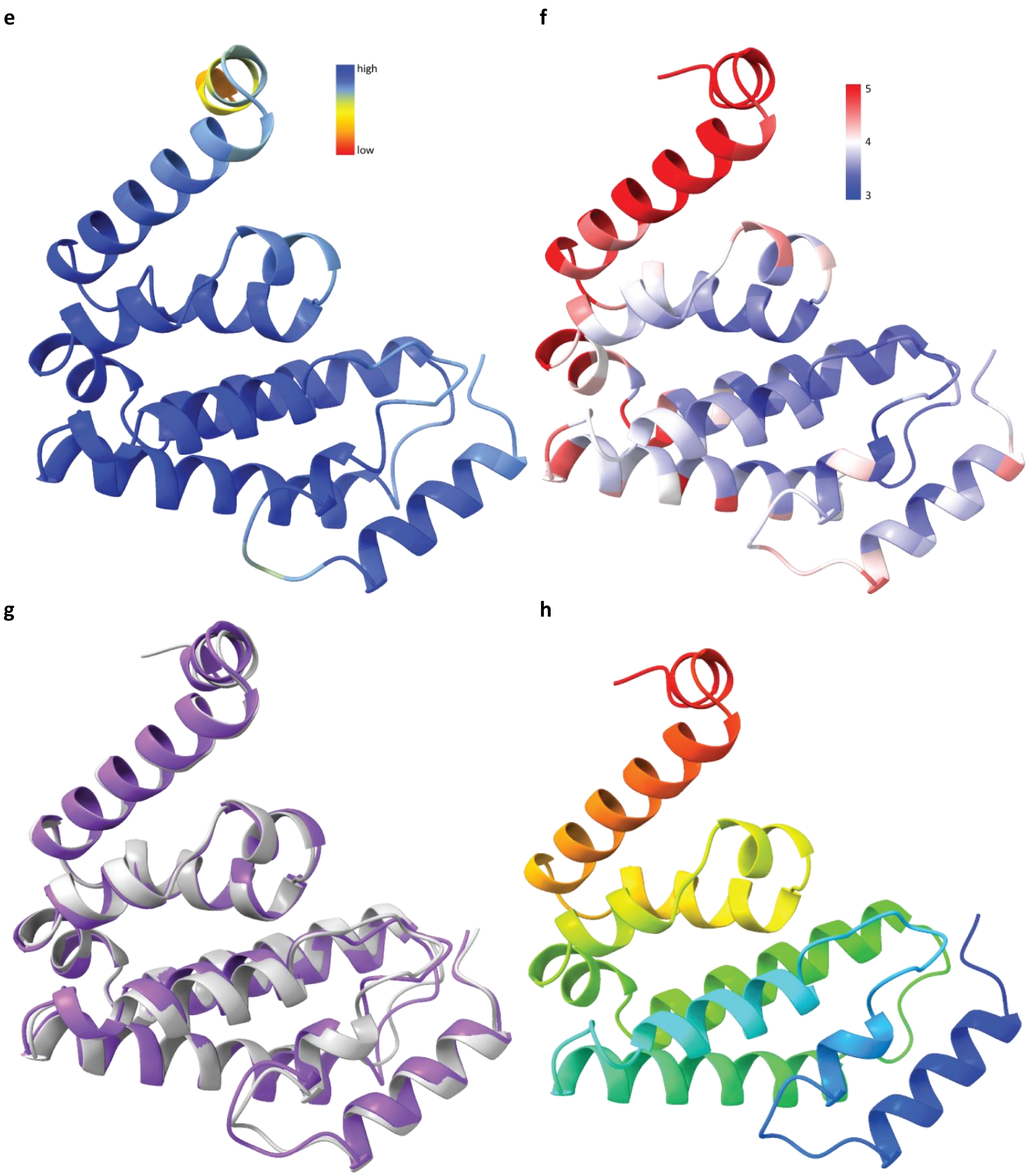
e
high
low
f
5
4
3
g
h

Supplementary Fig. 7 Models predicted by AlphaFold2 compared to the experimental model. a Predicted PAPP-A monomer atomic model generated in AlphaFold2 and colored according to pLDDT confidence interval values[2]. **b** Local resolution of the PAPP-A monomer experimental structure. Resolution values are interpolated from local resolution maps onto the atomic model (color bar indicates Å values). Local resolutions of MAP1 and MAP2 were used. **c** AlphaFold2-predicted structure (grey) of the PAPP-A monomer superimposed with experimental PAPP-A monomer (colored according to domains, as indicated). The alphafold2 model is trimmed to fit the sequence of the experimental model. Note the different directions of the SCR region. **d** PAPP-A monomer structure rainbow-colored with the N-terminal blue and C-terminal red. **e** Predicted STC2 monomer atomic model generated in AlphaFold2 and colored according to pLDDT confidence interval values. **f** Local resolution of the STC2 monomer experimental structure. Resolution values are interpolated from local resolution maps onto the atomic model (color bar indicates Å values). Local resolution of MAP1 were used. **g** AlphaFold2-predicted model (purple) of the STC2 monomer superimposed with the experimental STC2 monomer (grey). The alphafold2 model is trimmed to fit the sequence of the experimental model. **h** STC2 monomer structure rainbow-colored with the N-terminal blue and the C-terminal red.

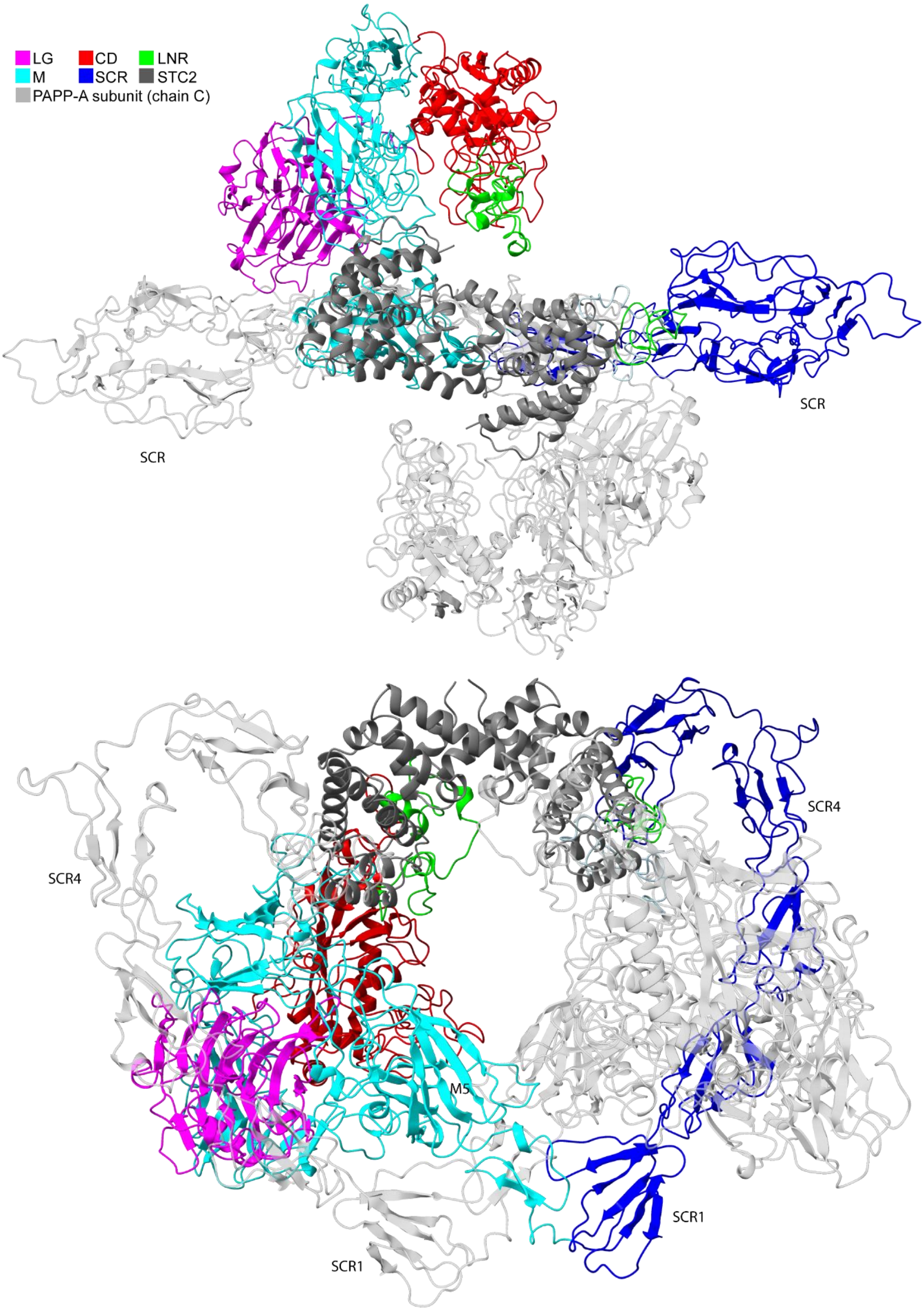

Supplementary Fig. 8 Relationship between subunits of the two PAPP-A monomers emphasized. Cartoon representation of the full PAPP-A·STC2 2:2 complex two different orientations (top view and side view as in Fig. 2c). Domains are colored as indicated, with one PAPP-A subunit (chain C) in light grey (transparent).

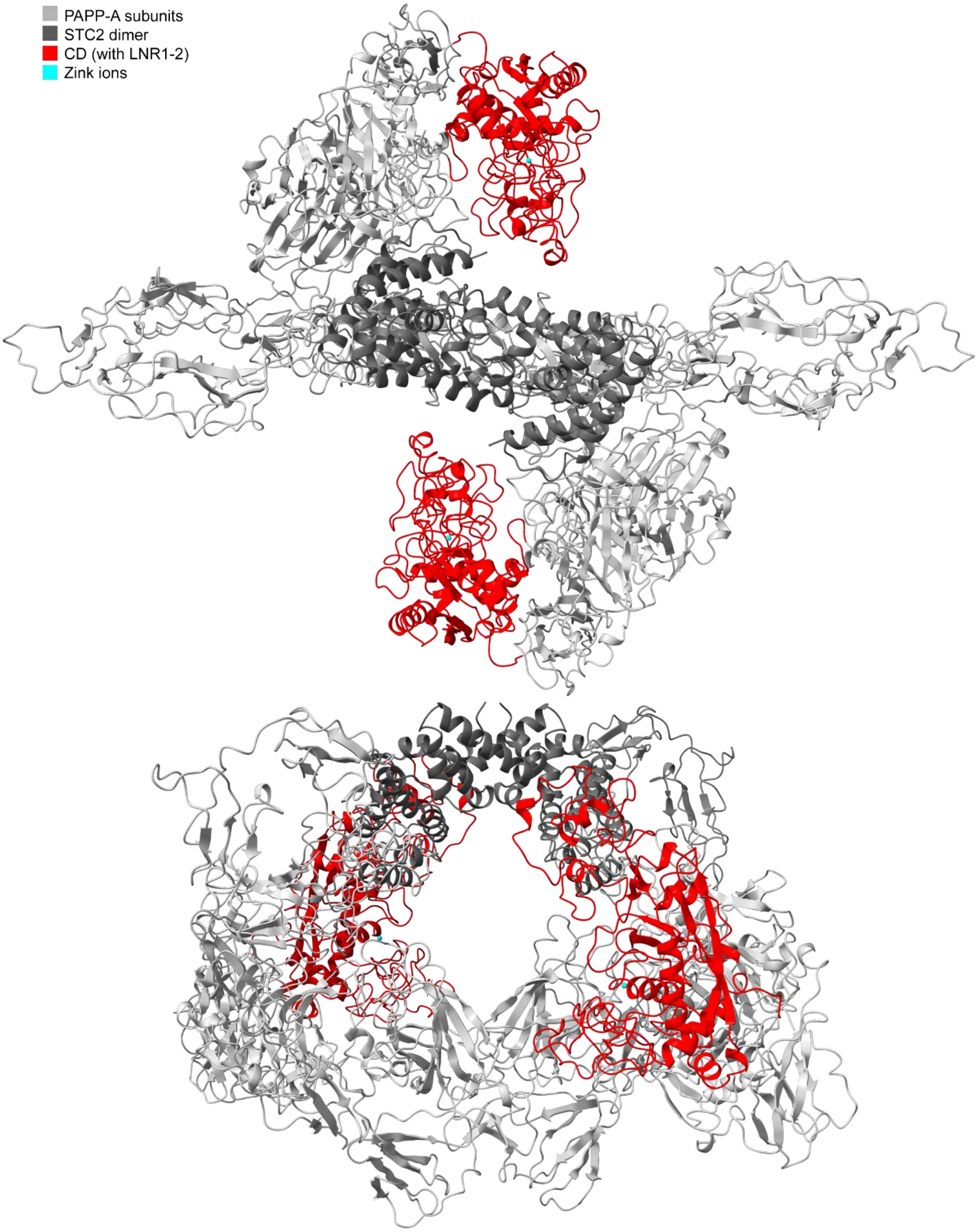

Supplementary Fig. 9 Cartoon representation of the PAPP-A·STC2 2:2 complex emphasizing the orientation of the active site of the CD. The STC2 dimer is colored dark grey, and both PAPP-A subunits are colored light grey, except for the CDs (including LNR1-2), which are colored red. In both CDs, the active site zinc ion is colored cyan. A top view (top) and a side view (bottom) are shown.

a

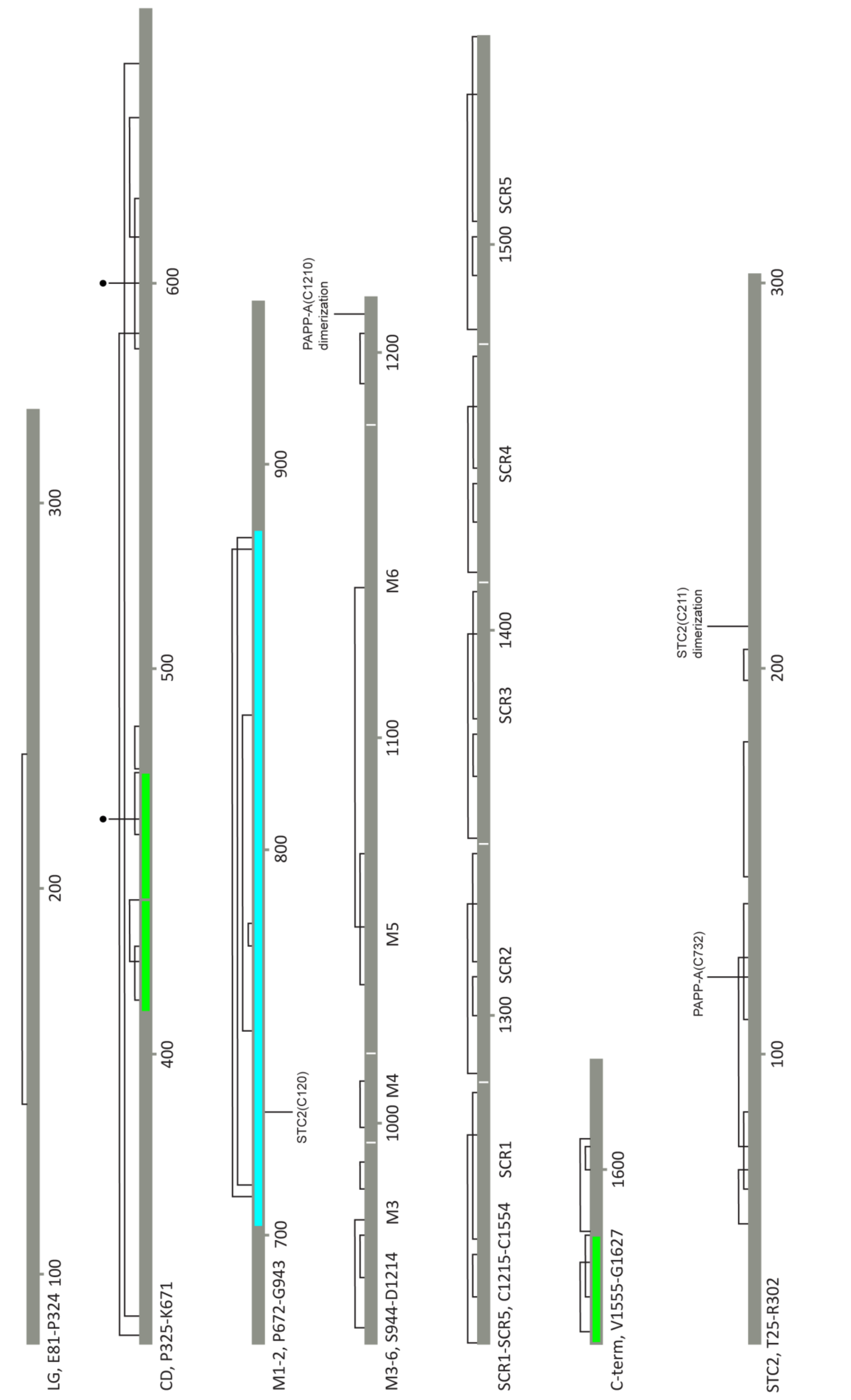
LG, E81-P324
CD, P325-K671
M1-2, P672-G943
M3-6, S944-D1214
SCR1-SCR5, C1215-C1554
C-term, V1555-G1627
STC2, T25-R302
100
200
300
400
500
600
700
800
900
1000
1100
1200
1300
1400
1500
1600
M3
M4
M5
M6
SCR1
SCR2
SCR3
SCR4
SCR5
STC2(C120)
PAPP-A(C1210) dimerization
STC2(C211) dimerization
PAPP-A(C732)

b

LG
C144-C235*
C235-C144*

CD
C327-C587*
C332-C657*
C414-C428*
C424-C440*
C428-C414*
C440-C424*
C457-C473
C461-UNPAIRED*
C473-C457
C474-C485*
C485-C474*
C583-C622*
C587-C327*
C600-UNPAIRED*
C612-C643*
C622-C583*
C643-C612*
C657-C332*

M1-M6
M2
C710-C881*
C713-C878*
C732-STC2(C120)*
C753-C835*
C775-C781*
C781-C775*
C835-C753*
C878-C713*
C881-C710*
M3
C947-C975*
C960-C971*
C971-C960*
C975-C947*
M4
C983-C990*
C990-C983*
C999-C1011*
C1011-C999*
M5
C1036-C1070
C1051-C1139
C1070-C1036
C1139-C1051
M6
C1192-C1205*
C1205-C1192*
C1210-PA DIM.*

SCR1-5
SCR1
C1215-C1269
C1227-C1238
C1238-C1227
C1242-C1280
C1269-C1215
C1280-C1242
SCR2
C1285-C1329*
C1300-C1310*
C1310-C1300*
C1314-C1342*
C1329-C1285*
C1342-C1314*
SCR3
C1346-C1399*
C1362-C1373*
C1373-C1362*
C1377-C1410
C1399-C1346*
C1410-C1377
SCR4
C1415-C1458*
C1428-C1438
C1438-C1428
C1442-C1471*
C1458-C1415*
C1471-C1442*
SCR5
C1478-C1539*
C1492-C1502
C1502-C1492
C1506-C1554*
C1539-C1478*
C1554-C1506*

C-TERMINAL
C1558-C1576*
C1567-C1583*
C1576-C1558*
C1583-C1567*

C1584-C1608*
C1600-C1606*
C1606-C1600*
C1608-C1584*

STC2
C56-C70*
C65-C85*
C70-C56*
C76-C125*
C85-C65*
C109-C139*
C120-PAPP-A(C732)*
C125-C76*
C139-C109*
C146-C181*
C181-C146*
C197-C205*
C205-C197*
C211-STC2 DIM.*

Supplementary Fig. 10 Status of cysteine residues of the PAPP-A·STC2 complex. a Schematic drawing of cysteine connectivity. Residues engaged in homo- and heterodimerization are indicated (one of each type in total in each subunit). The three LNR domains are shown in green, and the M2 domain is shown in cyan. Unpaired cysteine residues are indicated with a filled circle. The PAPP-A subunit contains 39 intrachain disulfides and two unpaired cysteines, the STC2 subunit contains six intrachain disulfides. **b** List of cysteine residues of the PAPP-A·STC2 complex with specification of pairing. Two residues are unpaired in the structure, as indicated. An asterisk indicate disulfide bonds experimentally confirmed in the density map. The remaining are inferred based on distance between backbone atoms.

a

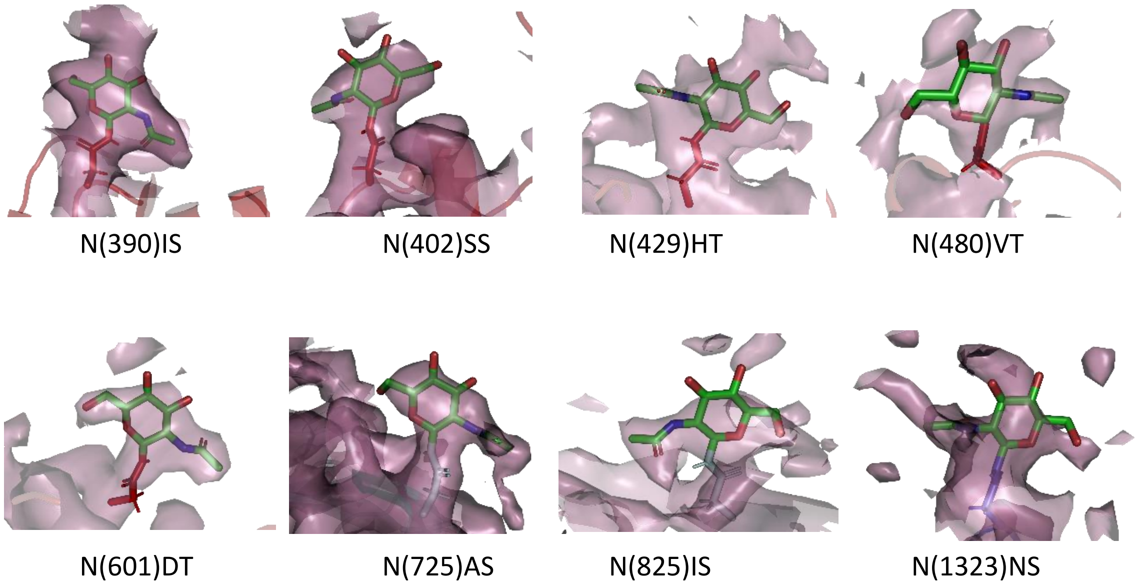
N(390)IS
N(402)SS
N(429)HT
N(480)VT
N(601)DT
N(725)AS
N(825)IS
N(1323)NS

b

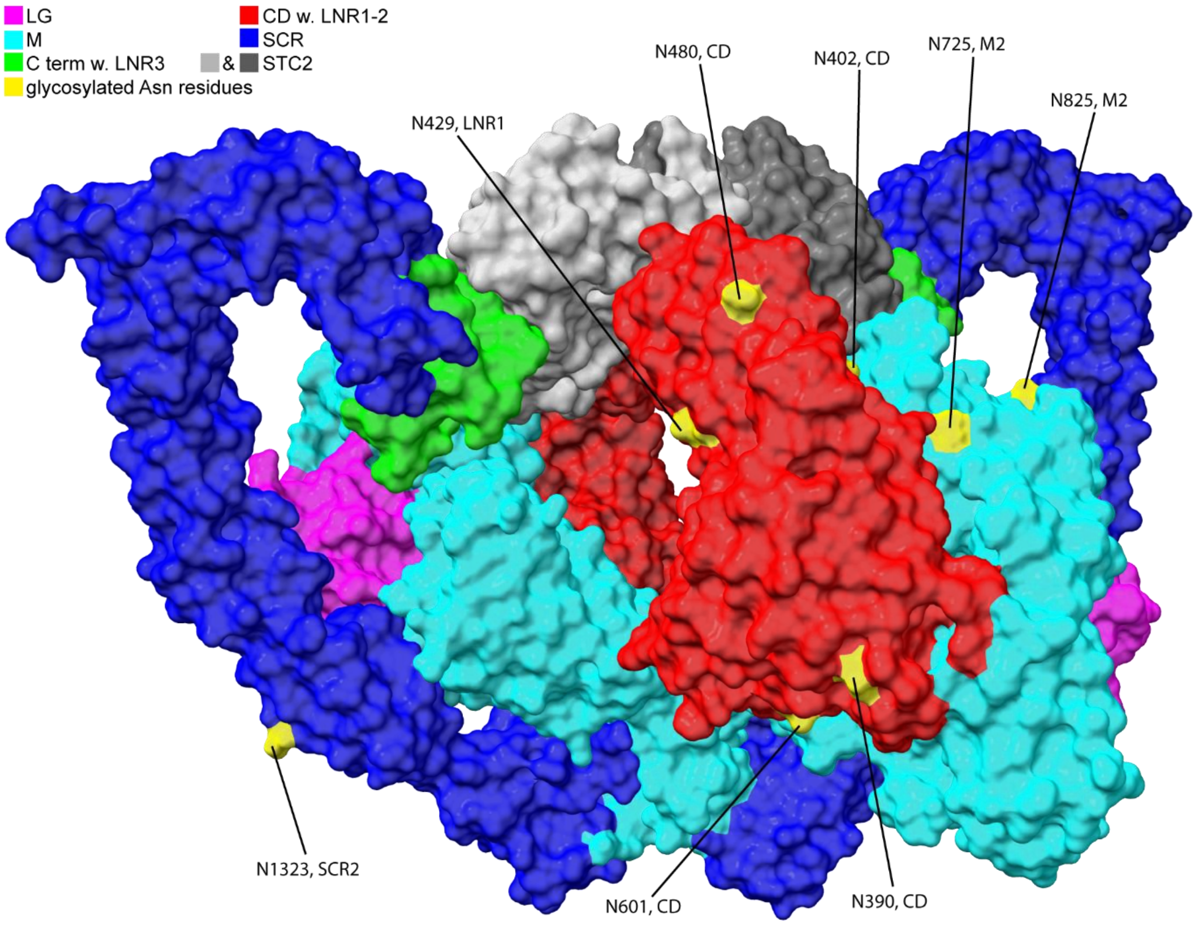
LG
M
C term w. LNR3
glycosylated Asn residues
CD w. LNR1-2
SCR
& STC2
N480, CD
N402, CD
N725, M2
N825, M2
N429, LNR1
N1323, SCR2
N601, CD
N390, CD

Supplementary Fig. 11 Occupancy of potential N-glycosylation sites of the PAPP-A and STC2 subunits. a Of 14 potential N-glycosylation sites (NX(S/T), (N(390)IS, N(402)SS, N(429)HT, N(480)VT, N(601)DT, N(619)DT, N(725)AS, N(825)IS, N(1026)AS, N(1222)AS, N(1226)CS, N(1323)NS, N(1465)GS, and N(1519)VT), we find evidence that eight are occupied in the recombinant material used in the current study. The total carbohydrate content of recombinant PAPP-A is approximately half[1] of the amount (13.3% (w/w)) found in the native material purified from human serum[3], in which 11 sites are occupied[3]. The potential N-glycosylation site of STC2, N(73)NS, was not occupied in the PAPP-A·STC2 complex. Enlarged views of the substituted asparagine residues, as indicated, are shown with density map. The map (MAP1) was contoured at 2-4 σ. **b** A surface representation of the PAPP-A·STC2 complex with colored domains showing positions of N-linked carbohydrates. Asparagine residues carrying carbohydrate are shown in yellow, and the corresponding residue numbers are indicated. Only occupied asparagine residues of one of the two PAPP-A subunits (the C chain) are shown. Domains are colored as indicated.

a

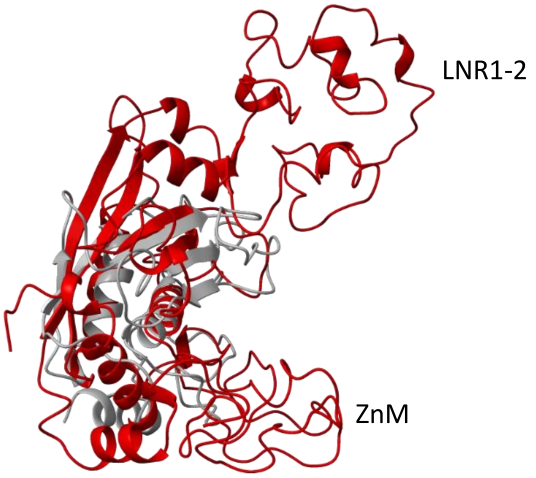

MMP12, RMSD = 1.22 Å, ZnM: 7 residues

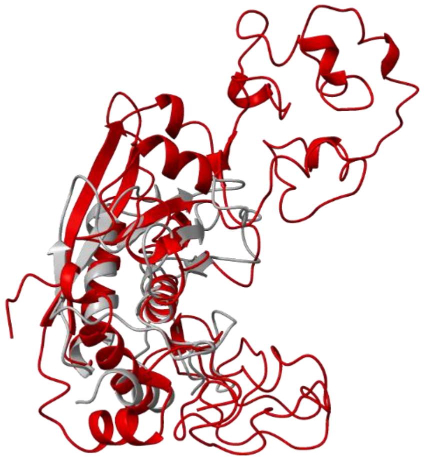

Matrilysin, RMSD = 0.82, ZnM: 7 residues

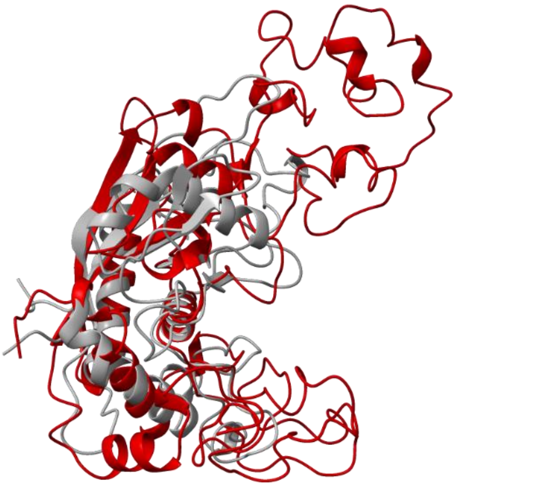

ADAM17, RMSD = 0.98 Å, ZnM: 19 residues

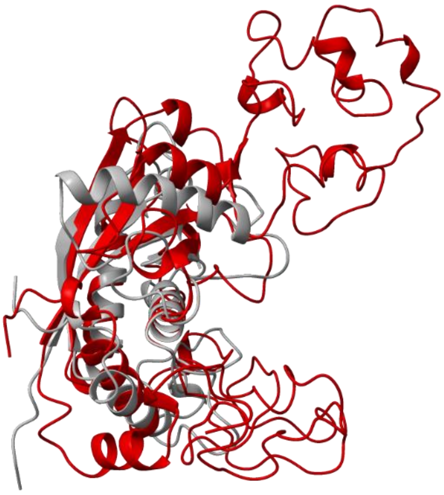

ADAM33, RMSD =1.15 Å, ZnM: 17 residues

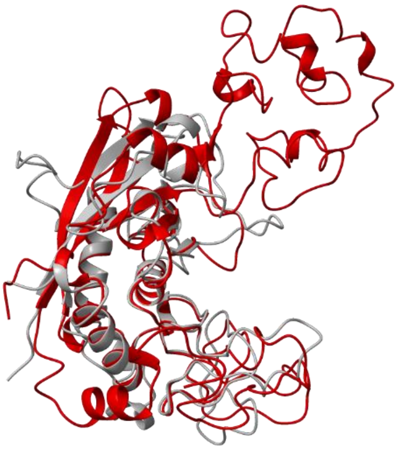

Ulilysin, RMSD = 0.90 Å, ZnM: 48 residues

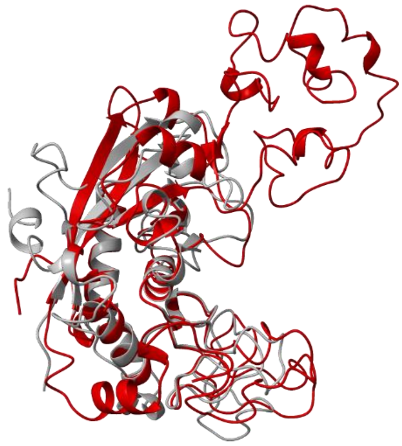

Mirolysin, RMSD = 0.91 Å, ZnM: 46 residues

b

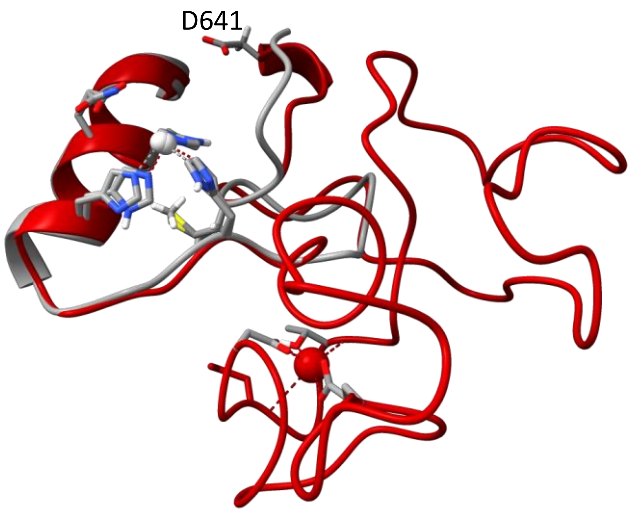

Matrilysin

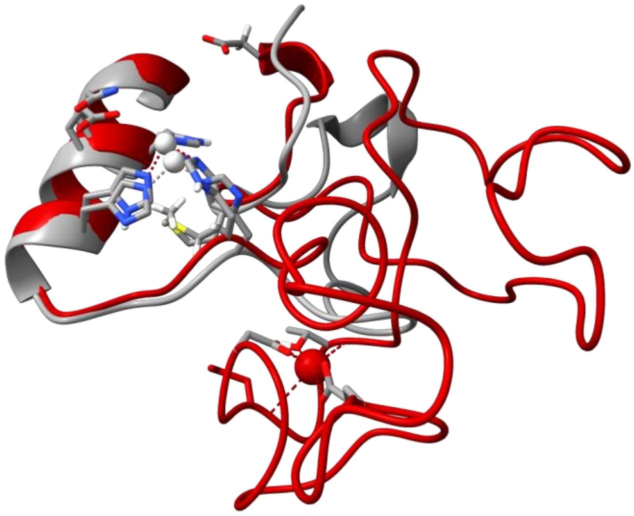

ADAM33

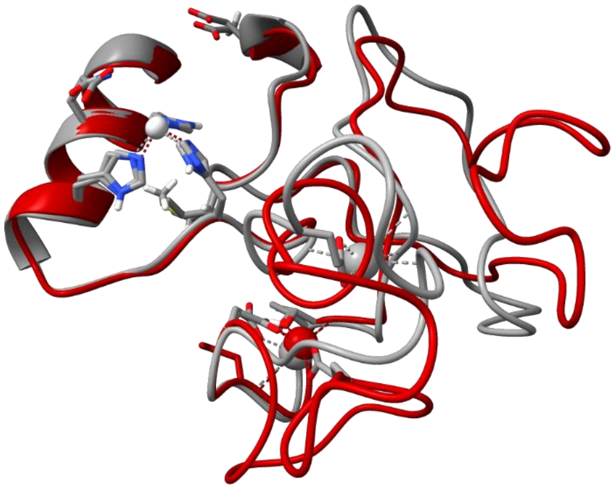

Ulilysin

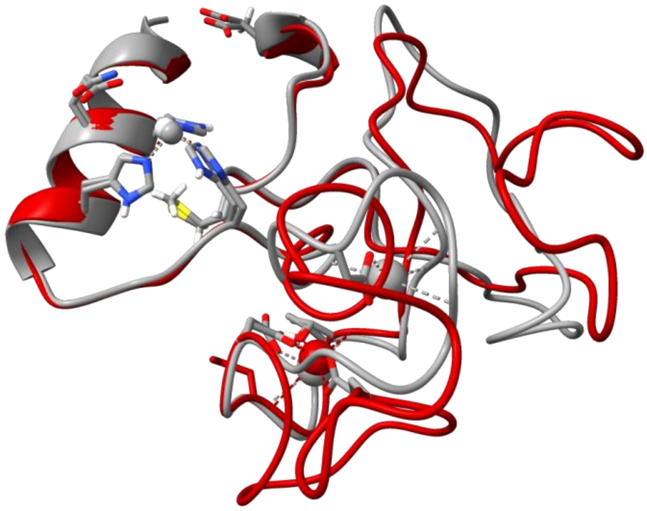

Mirolysin

Supplementary Fig. 12 Comparison of the PAPP-A CD with other representative metzincin metalloproteinases. a Superimpositions of the PAPP-A CD (red) and other (grey) metzincins of the matrix metalloproteinase family (MMP12 (4H49)[4] and matrilysin (1MMQ)[5]), the adamalysin family (ADAM17 (3G42)[6] and ADAM33 (1R54)[7]), and the pappalysin family (ulilysin (3LUM)[8] mirolysin (6R7W)[9]), as indicated in the panel. Active site clefts are facing towards the right. For each comparison, the backbone root-mean-square deviation (RMSD) and the number of residues of the ZnM loop is also indicated. The position of the LNR modules and the ZnM loop is indicated (top left). **b** Comparison of the active site environment of PAPP-A with that of matrilysin, ADAM33, ulilysin, and mirolysin, as indicated. PAPP-A residues H558-D642 of the CD are shown (red), corresponding to the α-helix of the active site followed by the ZnM loop. The tree histidine residues and the Zn^{2+} ion they coordinate, the methionine residue of the Met-turn (replaced with a leucine in ulilysine), and the active site glutamic acid (replaced with a glutamine in PAPP-A) above the Zn^{2+} ion are shown for all structures. The single Ca^{2+} ion of the PAPP-A ZnM loop (red) and the two Ca^{2+} ions of ulilysin and mirolysin (grey) are shown with coordinating residues. Note that residues corresponding to PAPP-A residue D641 (indicated top left) are present only in ulilysin and mirolysin.

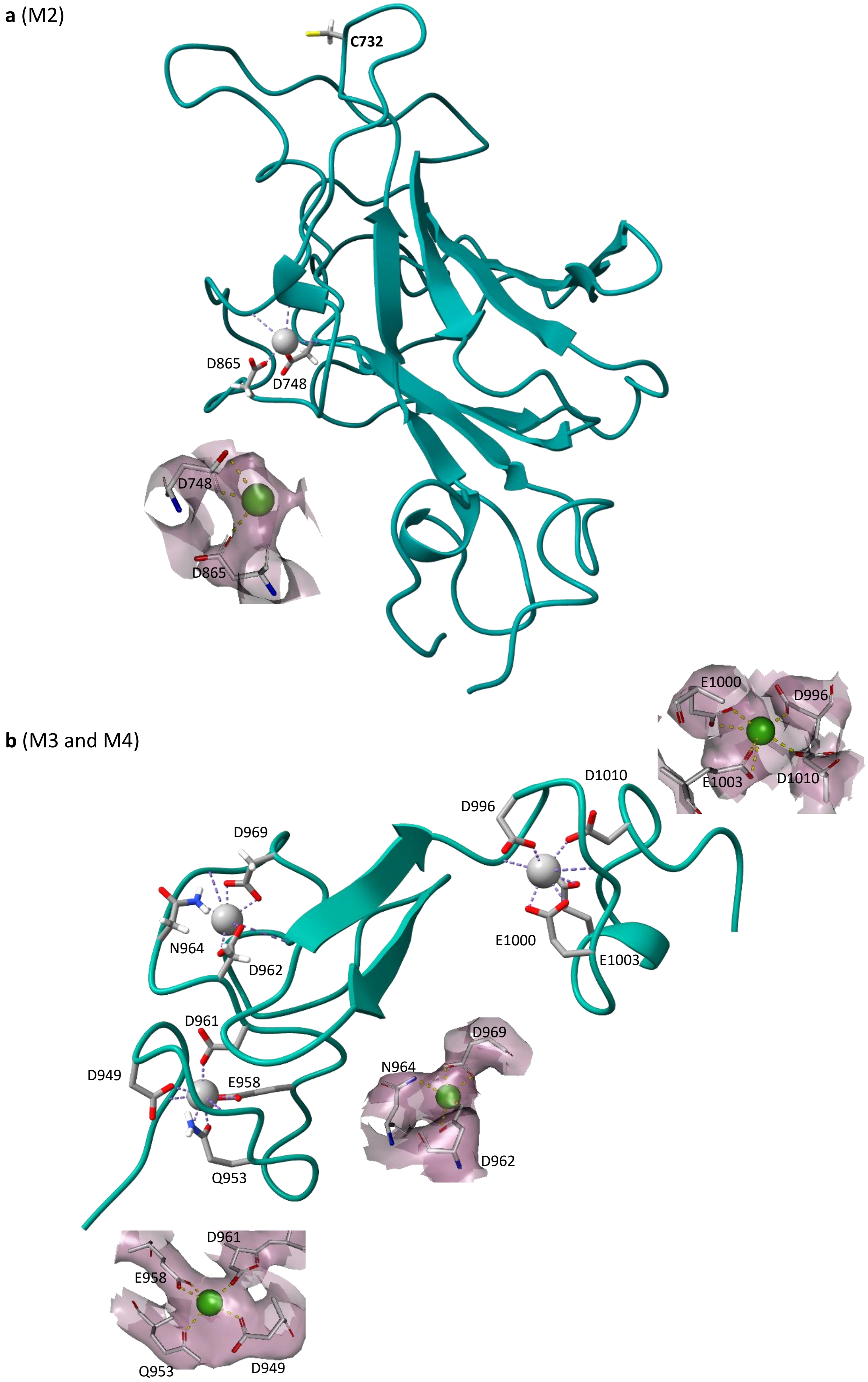
a (M2)
C732
D865
D748
D748
D865
b (M3 and M4)
E1000
D996
E1003
D1010
D1010
D996
D969
N964
D962
E1000
E1003
D961
D949
E958
Q953
D969
N964
D962
D961
E958
Q953
D949

c (M2 and M5)

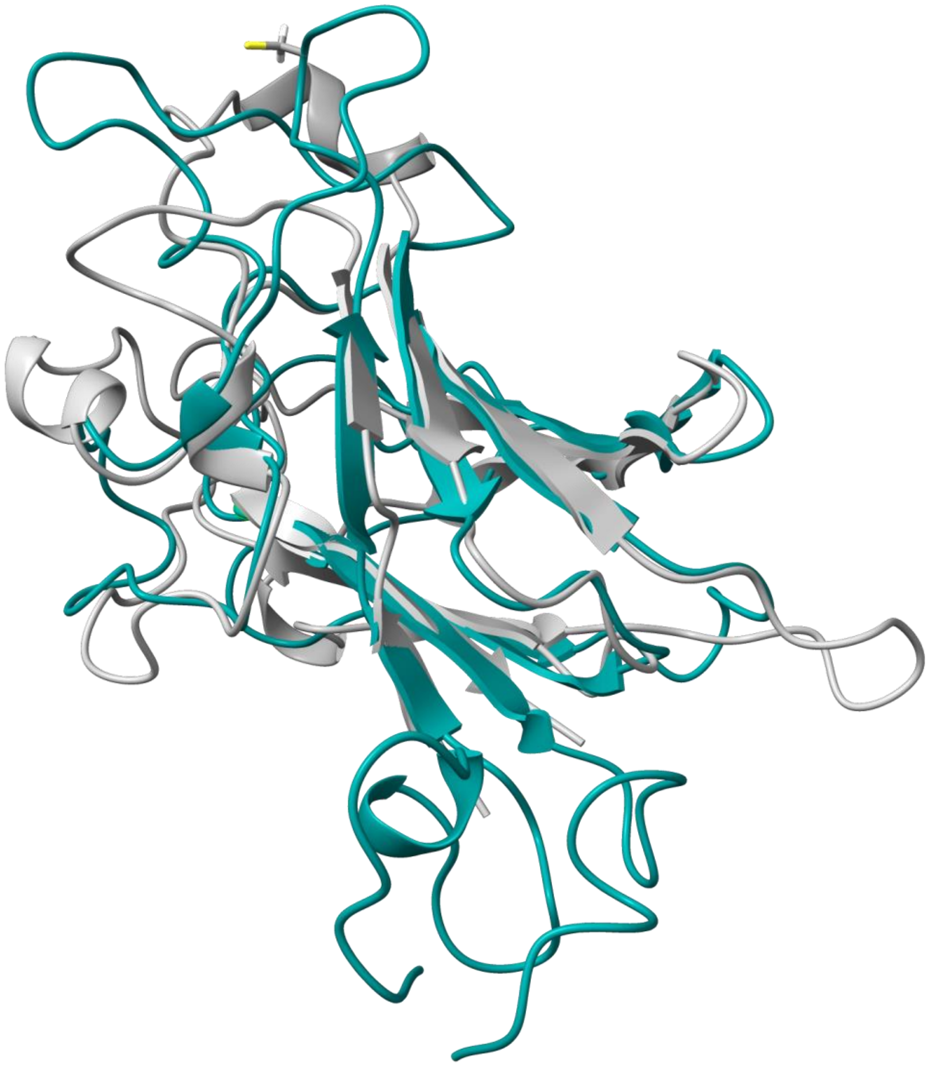

Supplementary Fig. 13 PAPP-A M domains: Calcium ion binding and comparison of M2 and M5. a The structure of M2 (F702-P883) with bound Ca^{2+} ion and coordinating residues is shown. The position of C732 (which binds to C120 of STC2) is indicated. Insets show map around the Ca^{2+} ions. The map (MAP1) was contoured at 4 σ. **b** Similar to b, but for partial structure corresponding to M3 (left side) and M4 (S944-Y1014). The map (MAP1) was contoured at 4-5 σ. **c** Domains M2 (cyan) and M5 (grey), both fibronectin-iii type domains, superimposed (RMSD = 0.99 Å).

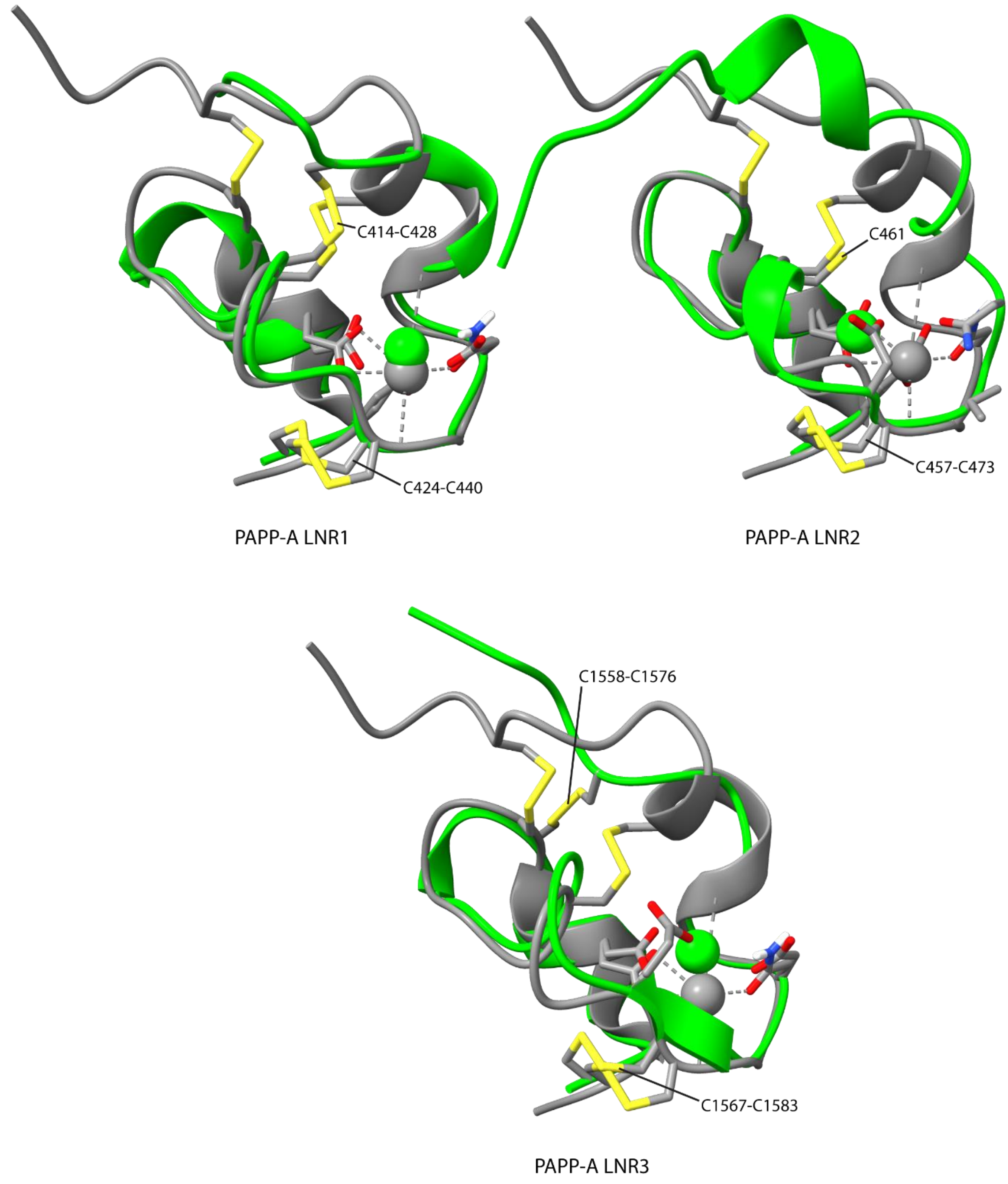

Supplementary Fig. 14 Comparison of PAPP-A LNR modules with Notch1 LNR. Superimposition of PAPP-A LNR domains (green, LNR1, LNR2, and LNR3, as indicated) and the LNR_A module of human Notch1 (grey)[10] (1PB5, model 1.1). The three disulfide bonds of the Notch LNR modules and the two (LNR1 and LNR3) or one (LNR2) disulfide bonds of the PAPP-A LNR modules are shown.

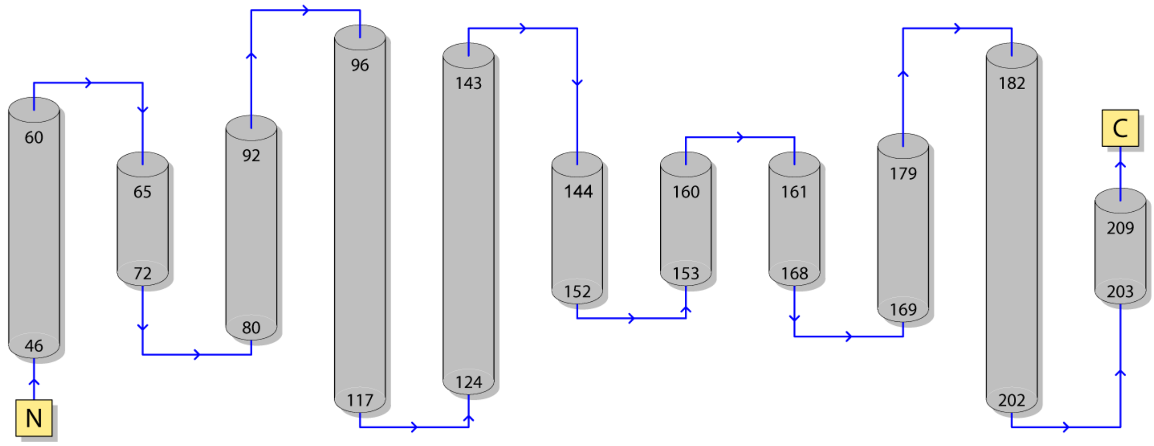

Supplementary Fig. 15 Topology diagram of the STC2 monomer generated in PDBsum[11]. STC2 consist of 11 α-helices. The start and end residue of each element are indicated.

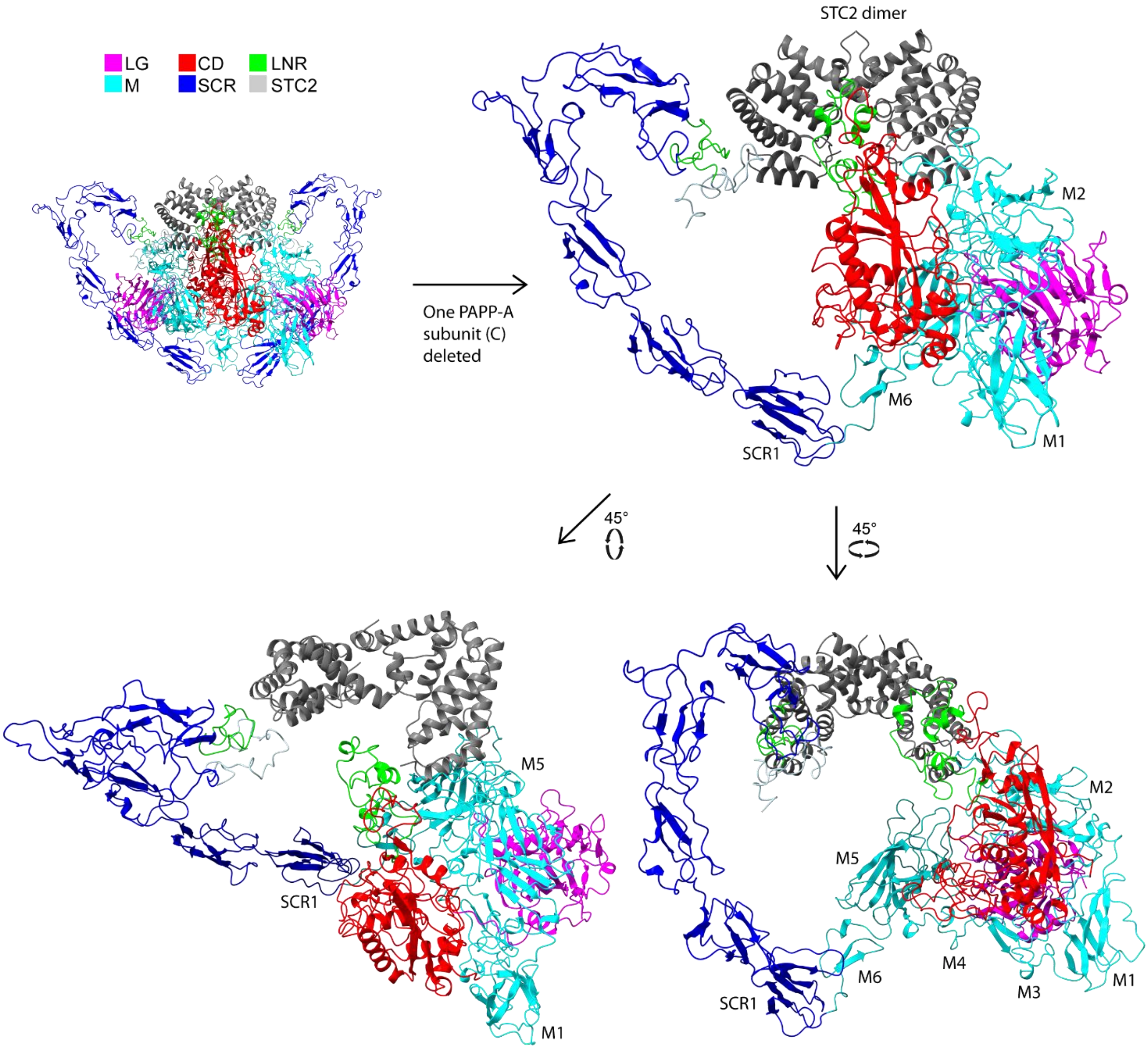

Supplementary Fig. 16 Position of the STC2 dimer relative to the PAPP-A monomer. Cartoon representation of the full PAPP-A·STC2 2:2 complex is shown (top left, similar to Fig. 2c), and the same structure is shown without one of the two PAPP-A subunits (chain C is deleted, chain Q is shown). The latter is shown in two additional orientations. Domains are colored as indicated.

a

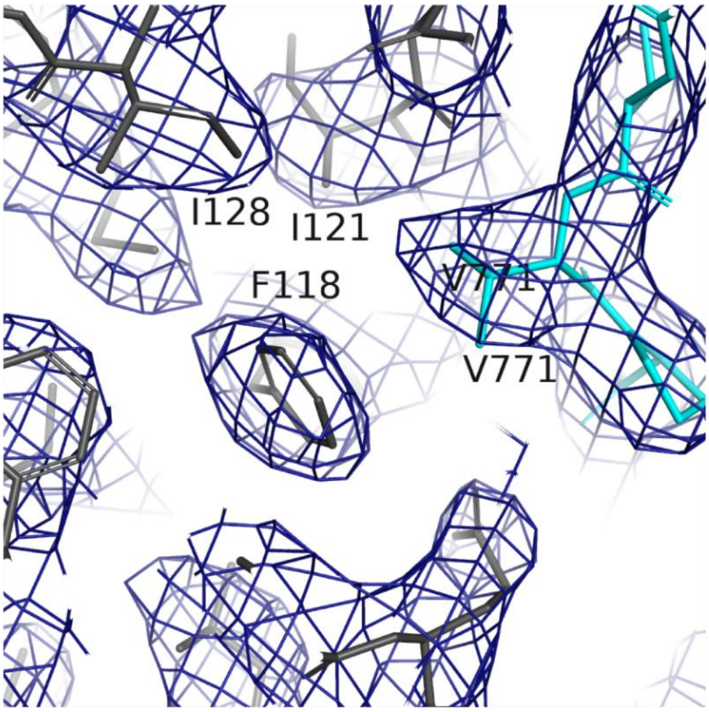

b

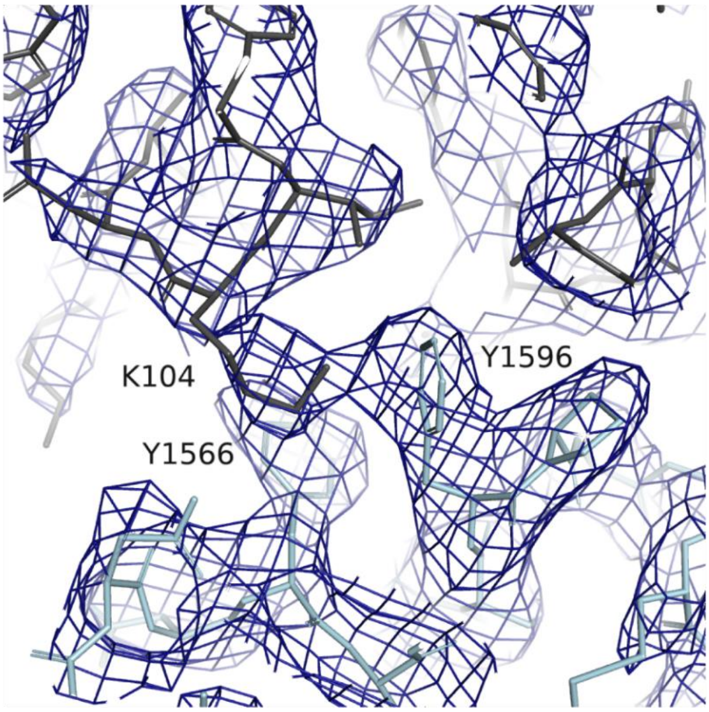

Supplementary Fig. 17 Focused views to illustrate MAP1 density at interfaces between PAPP-A and STC2. a Density of central residues in the interface between the M1 region of PAPP-A (cyan) and STC2 (grey). The map (MAP1) was contoured at 5.0 σ. **b** Density of central residues in the interface between the C-terminal of PAPP-A (light blue) and STC2 (grey). The map (MAP1) was contoured at 5.0 σ. Note that this represents an alternative view compared to Figure 7c.

a

```
Human_PA        -----------------------------------------------MRLWSWVLHLGLL   13
Mouse_PA        -----------------------------------------------MRLWSWVLRLGLL   13
Dog_PA          -----------------------------------------------MRLWSWVLRLGLL   13
Chicken_PA      MAGGTPSVLHWLQWGCRAGRKSRGAASDCLDVRCVHGGSMSHCTSYLAVEEHIRPSLKSL   60
Frog_PA         -----------------------------------------------MLLWSLLLPLGLL   13
Zebraf_PAa      -----------------------------------------------MKVWTFLQCLAIL   13
Zebraf_PAb      ---------------------------------------------MKLLPICTWLLCL--L   14
                                                        *  *

Human_PA        ----SAALGCGLAERPRRARRDPRAGRPPRPAAGPATCATRAARGRRASPPP--PPPPGG   67
Mouse_PA        ----SAALGCGLAERPRRVRRDPRAVRPPRPAAGPATCATRAARGRRASP----PPPPGG   65
Dog_PA          ----SAALGCGLAERPRRARRDPRPGRPPRPAAGPATCATRAARGRRASPPPPPPPPPGG   69
Chicken_PA      ----ASGPECGMDERSRRARRDTRHSRQLL-YTAPGTCATRLARGRRSTAGL--------   107
Frog_PA         CAALASASQCGMDERSQRPRRNVRNPRSLR-RGSEGSCATSLARGRRSLPSI--------   64
Zebraf_PAa      LVLLCVGSECGTVLRNTRSKRELVKIREAK-LVFPGACGTRLPRGKRSLPGM--------   64
Zebraf_PAb      LFLVCLGSECGTPRRIVRSKRDLVRIREAR-STFPGACATRLPRGKRSLPGI--------   65
                    . .  **   *  * :*:    *        .:*.*   **:*:

                              >-start of mature PAPP-A
                              >-Laminin G-like (E81-P324)
Human_PA        AWEAVRVPRRRQQREARGATEEPSPPSRALYFSGRGEQLRLRADLELPRDAFTLQVWLRA   127
Mouse_PA        AWEAVRVPRRRQQRAARGAE-EPSPPSRALYFSGRGEQLRLRADLELPRDAFTLQVWLRA   124
Dog_PA          AWEAVRVPRRRQQREARGAA-EPGPPGRALYFGGRGEQLRLRADLELPRDAFTLQVWLRA   128
Chicken_PA      --EPGHVPRRRQQREVEDGE-ESLTPSRALYFSGQGDQLRLKADIELPRDAFTLQVWLKA   164
Frog_PA         --EHLRIPQRRKQREAKESS-NVPAPGKALYFTGHGDQLRLKSGNELPRDTFTLQVWLRA   121
Zebraf_PAa      ---DRRIPRQRRRSSPTED--ASSSRGKAVYFTGRGDQLRLKPGVEIPKGNFTLQMWVKA   119
Zebraf_PAb      ---DRRSPQLSQAGE------SSPGRRRAVYFTGRGDQLRLKPNAEVPRANFTLEMWIKP   116
                     : *:  :               :*:** *:*:****: . *:*:  ***::*::

Human_PA        EGGQRSPAVITGLYDKCSYISRDRGWVVGIHTISDQDNKDPRYFFSLKTDRARQVTTINA   187
Mouse_PA        EGGQKSPAVITGLYDKCSYTSRDRGWVMGIHTTSDQGNRDPRYFFSLKTDRARKVTTIDA   184
Dog_PA          EGGQRSPAVITGLYDKCSYTSRDRGWVVGIHTVSDQGNRDPRYFFSLKTDRARQVTTINA   188
Chicken_PA      EGGQRSPAVIAGLYDKCSYTSRDRGWVLGINTVSDQGNRDPRYFFSLKTDRARKVTTIAA   224
Frog_PA         EGGQKSPAVIAGLYDKCSYTSHDRGWVLGIEALSDQGTRDPRYFFTLRTDRARKATTITA   181
Zebraf_PAa      EGGQRSPTVIAGLFDKCFYASSDRGWLLGIKSVSEQGNRDPRFFFSLKTDRAHKVASIHS   179
Zebraf_PAb      EGGQRSPAVIGGLYDKCFYASSDRGWLFGVKAVSDQANRDPRFFFSLKTDRAHKLTTITS   176
                ****:**:** **:*** * * ****:.*:.: *:* .:***:**:*:****:: ::* :

Human_PA        HRSYLPGQWVYLAATYDGQFMKLYVNGAQVATSGEQVGGIFSPLTQKCKVLMLGGSALNH   247
Mouse_PA        HRSYLPGQWVHLAATYDGRLMKLYMNGAQVATSAEQVGGIFSPLTQKCKVLMLGGSALNH   244
Dog_PA          HRSYVPGQWVHLAATYDGRLMKLYVNGAQVATSGEQVGGIFSPLTQKCKVLMLGGSALNQ   248
Chicken_PA      HRSYLPNQWVHLAATYDGHLMKLYVNGAQVATSGEQVGSIFSLLTLKCKVLMVGGNALNQ   284
Frog_PA         HRSYLPNQWVHLAVTYNGRIIKLYVNGAQAATSNEQVGPIFSPLTQKCKVLMIGGNAQNQ   241
Zebraf_PAa      NARYLPNHWVHVAVTYNGLFMKLYINGAQTAVSREQSGDVFSPLTKKCKVLMVGGNALNH   239
Zebraf_PAb      NTAYTPNQWSHVAVTYNGLFMKLYVNGAQVAVSRDQSGEIFSPLTKKCKVLMIGGNALNH   236
                :  * *.:* ::*.**:* ::***:****.*.* :* * :** ** ******:**.* *:

Human_PA        NYRGYIEHFSLWKVARTQREILSDMET--HGAHTALPQLLLQENWDNVKHAWSPMKDGSS   305
Mouse_PA        NFRGHIEHFSLWKVARTQREIVSDMET--RGLHTPLPQLLLQENWDNVKRTWSPMKDGNS   302
Dog_PA          NFRGYIEHLSLWKVARTQREVLLDMES--HGLHAPLPQLLLQENWDNVKRTWSPMKDGHM   306
Chicken_PA      NYRGYVEHFSLWRTARSQKEILLDMGQAIHRQDMPLPQLVLQDSLLNVKNTWSPMKDGSS   344
Frog_PA         NYRGYLEQFTLWKTPRTQEKIVHDMGQAIHGLSTSLPQLVLQDSFENVKHAWSPMKEGKF   301
Zebraf_PAa      NYRGSVERLGLWRQALSQRQIMRDMHGRED-P-EDLVDLVIRETFEHPARKWLAVKDGSF   297
Zebraf_PAb      NYRGTMEHLSLWHQALTQREIIRSMRQGLAQL-VDLSQLVIHENFENMSRRWLTVKDGSF   295
                *:** :*:: **:   :*.::: .*          * :*::::.  :  . *  :*:*

                                     >-CD (P325-K671)
Human_PA        PKVEFSNAHGF--LLDTSLEPPLCGQTLCDNTEVIASYNQLSSFRQPKVVRYRVVNLYED   363
Mouse_PA        PQVEFSNAHGF--LLDTNLEPPLCGQTLCDNTEVISSYNQLPSFRQPKVVRYRVVNIYDD   360
Dog_PA          PHVEVSGAHGF--LLDTSLEPPLCGQTLCDNTEVIASYNQLPRFRRPKVVRYRVVNLRDD   364
Chicken_PA      PQSKSSYHHGY--LLDTSLEPPLCGQTVCDNTDVIASYNKLPSFRRNKIVRYRVVNLYDD   402
Frog_PA         PQIENIYHHGS--SLDTILDLPQCGQTLCDNLEVITNYNKFTSFRQPKVVRYRVVNVYDD   359
Zebraf_PAa      PIPDDGPRAGLG-SLDTTLEPPACGQTVCDNTEVVRNYNHFWSFRQPKTVRYRVVNVFDD   356
Zebraf_PAb      PRLEHSDRSLASDLSDSALKPPACGQTVCDNVEVITNYYQLWSFRKPKTVRYRVINVYDD   355
                *  .           *: *. * ****:*** :*: .* ::  **: * *****:*: :*
```

```
                                                                 >-LNR1-------
Human_PA        DHKNPTVTREQVDFQHHQLAEAFKQYNISWELDVLEVSNSSLRRRLILANCDISKIGDEN   423
Mouse_PA        HHENPTVSWQQIDFQHQQLAEAFQHYNISWELEVLNINSSSLRHRLILANCDISKIGDEK   420
Dog_PA          DRENPTVSRQQIDFQHRQLADAFKHYNISWELQVLEVSNSSLRRRLVLANCDISKIGDEN   424
Chicken_PA      KHQNPTVSQEQIEFQHQHLNEAFSRYNITWELEVLEVKNSSLRHRLILANCDISKIGDEN   462
Frog_PA         NHENPTVTKDQIELQHRKLNEAFSKYNITWELDLLEKNDSFLRHRLILTNCDITKIGDGF   419
Zebraf_PAa      FQRKPTVTDQQIHLQHQHLNEAFSMYNISWELTVHNVSNSSLYNRLVLANCDISKVGDDD   416
Zebraf_PAb      DGRFPTITEHQINLQHQHLNNAFRVYNITWERTVHNVYNSSLRNRLILANCDVSKVGDEE   415
                  . **:: .*:.:**::* :**  ***:**  : :  .* * .**:*:***::*:**

                ----------------<>-LNR2------------------------------<
Human_PA        CDPECNHTLTGHDGGDCRHL----RHPAFVKKQHNGVCDMDCNYERFNFDGGECCDPEIT   479
Mouse_PA        CDPECNHTLTGHDGGDCRQL----RYPAFMKKQQNGVCDMDCNYERFNFDGGECCDPDIT   476
Dog_PA          CDHECNHTLTGHDGGDCRNL----RHPAFVKKQHNGVCDMDCNYERFNFDGGECCNPEIT   480
Chicken_PA      CDPECNHTLTGYDGGDCRHV----RHTLFNKKKQNGVCDMDCNYERYNFDGGECCNPEIT   518
Frog_PA         CEPECNHALTGFDGGDCRRT----IPSVALRKKQNGVCDMDCNIESFHFDGGDCCNPNVT   475
Zebraf_PAa      CDPECNHTLTGFDAGFCKPRPEKHRTECPERKQGNGVCDPECNCENYNYDNGDCCNSSVT   476
Zebraf_PAb      CDPECNHTLTGFDAGFCKRQ----IARCPENKQGNGVCDPECNWDNFYYDHGDCCNPNIT   471
                *: ****:***.*.* *:            .*: ***** :** : : :* *:**: .:*

Human_PA        NVTQTCFDPDSPHRAYLDVNELKNILKLDGSTHLNIFFAKSSEEELAGVATWPWDKEALM   539
Mouse_PA        DVTKTCFDPDSPHRAYLDVNELKNILRLDGSTHLNIFFANSSEEELAGVATWPWDKEALM   536
Dog_PA          DVTKTCFDPDSPHRAYLDVNELKNILRLDGSTHLNIFFANSSEEELAGVATWPWDKEALM   540
Chicken_PA      EVTKTCFDPYSPYRAYLDVNELKNILKLDGSTHLNIFFANSSEEELAGVATWPWDKEALM   578
Frog_PA         DVTKTCFDPESPNRAYLDVNEMKNRLNLTGSTQLNIFFANSSE-ELAGVATWPWDKEVLT   534
Zebraf_PAa      DVTKTCFNPTSHLRAYLDVKELKEILKLDGSTHLNIFFANSSDEDLAGVATWPWDKDALA   536
Zebraf_PAb      DVTKTCFNPASPLRAYMNVKELKEVLNLDGSTHLNIFFANSSDEDVAGVATWPWDKEALT   531
                :**:***:* *  ***::*:*:*: *.* ***:******:**: ::**********:.*

                                      Zn binding
Human_PA        HLGGIVLNPSFYGMPGHTHTMIHEIGHSLGLYHVFRGISEIQSCSDPCMETEPSFETGDL   599
Mouse_PA        HLGGIVLNPSFYGIPGHTHTMIHEIGHSLGLYHIFRGISEIQSCSDPCMETEPSFETGDL   596
Dog_PA          HLGGIVLNPSFYGIPGHTHTMIHEIGHSLGLYHIFRGISEIQSCSDPCMETEPSFETGDL   600
Chicken_PA      HLGGIVLNPSFYGIPGHTHTMIHEIGHSLGLYHVFRGISEILSCSDPCMETEPSFETGDL   638
Frog_PA         HLGGIVMNPSFYGVPGHTHTMIHEIGHSLGLYHVFRGISEIQSCSDPCMETDPSFETGDL   594
Zebraf_PAa      HLGGIVLNPSFYGTFGHTHTMIHEIGHSLGLYHVFRGISEIESCNDACLETEPSLETGDL   596
Zebraf_PAb      HLGGIVLNPSFYGTFGHTHTMIHEIGHSLGLYHVFRGISEVESCNDACLETEPSLETGDL   591
                ******:******  ****************:******: **.* *:**:**:*****

                                                     Met-turn
Human_PA        CNDTNPAPKHKSCGDPGP--GNDTCGFHSFFNTPYNNFMSYADDDCTDSFTPNQVARMHC   657
Mouse_PA        CNDTNPAPKHKFCGDPGP--GNDTCGFHGFFNTPYNNFMSYADDDCTDSFTPNQVSRMHC   654
Dog_PA          CSDTNPAPKHKFCGDPGP--GNDTCGFHSFFNTPYNNFMSYADDDCTDSFTPNQVARMHC   658
Chicken_PA      CRDTNPAPKHKLCGDPGP--GNDTCGFHSFLNTPFSNFMSYADDDCTDSFTPNQVARMHC   696
Frog_PA         CRDTNPAPKHKMCGDPGPASGNDTCGFQKFVNTPFNNYMSYADDDCTDSFTPNQVARMHC   654
Zebraf_PAa      CADTNPTPKYKLCKDPEP--GNETCGNRNFVHTPFNNYMSYAEDDCTDSFTLNQVARMHC   654
Zebraf_PAb      CADTNPTPKYKYCSDPEP--GNDTCGRRHFTNTPFNNYMSYADDVCTDSFTLNQVARMHC   649
                * ****:**:* * ** *  **:*** : * :**:.*:****:* ****** ***:****

                       ---CD-<>-M1 (P672-F701+L884-G943)    >-M2 (F702-P883)
Human_PA        YLDLVYQGWQPSRKPAPVALAPQVLGHTTDSVTLEWFPPIDGHFFERELGSACHLCLEGR   717
Mouse_PA        YLDLVYQSWQPSRKPAPVALAPQVVGHTMDSVMLEWFPPIDGHFFERELGSACDLCLEGR   714
Dog_PA          YLDLVYQSWQPSRKPAPVALAPQIVGHTTNSVTLEWFPPIDGHFFERELGSACELCLEGR   718
Chicken_PA      YLDLVYQSWQPAKKPAPVAIAPQIVARTPTSVTLEWFPPIDGHFFEREVGSACDLCMEGR   756
Frog_PA         YLDLVYQSWQPTLKPLPVAIAPQIVERTPASVTLEWFPPIDGLFYEREVGTACHLCADQR   714
Zebraf_PAa      YLDLIYQTWQPNYRPPPVPMAPQVVEQDHGSISIEWFPPISGHFYDREVGSVCDKCAEGR   714
Zebraf_PAb      YLDLIYQSWRPGSKPAPVPLPPRVTAQDQHSLTLEWFPALTGHYLDREVGSVCDKCTEVG   709
                ****:** *:*  :* ** : *::  :   *: :**** : * : :**:*:.*. * :
```

```
                            C732                                  [766-777]
Human_PA        ILVQYASNASSPMPCSPSGHWSPREAEGHPDVEQPCKSSVRTWSPNSAVNPHTVPPACPE   777
Mouse_PA        ILVQYAFNASSPMPCGPSGHWSPREAEGHPDVEQPCKSSVRTWSPNSAVNPHTVPPACPE   774
Dog_PA          ILVQYAINASSPMPCGPSGHWSPREAEGHPDVEQPCKSSVRTWSPNSAVNPHTVPPACPE   778
Chicken_PA      VLVQHAFSASSPMPCDPSGHWSPREAEGHPDVEQPCKSSVRTWSPNSAVNQHTVPPACPE   816
Frog_PA         VLVQYAANASSPVPCNPSGHWSPREAEGHPDVEQPCETSVRTWSPNSGGNHHNVPPTCPE   774
Zebraf_PAa      ILLQYASNSSSPRPCSPSGHWSPREAEGPPDVEQACEPSVHTWSPHNGMDLGLVPPP-CP   773
Zebraf_PAb      VLLQYASNSSSPRPCAPSGHWSPREAEGPPDVEQACEASVSTWSPNAGLDSSSVGSASCP   769
                :*:*:*  .:*** ** ************ ***** *:  ** ****:  .  :    *

Human_PA        PQGCYLELEFLYPLVPESLTIWVTFVSTDWDSSGAVNDIKLLAVSGKNISLGPQNVFCDV   837
Mouse_PA        PQGCYLELEFRYPLVPESLTIWVTFVSSDWDSSGAVNDIKLLTISGKNISLGPQNVFCDI   834
Dog_PA          PQGCYLELEFRYPLVPESLTIWVTFVSTDWDSSGAVNDIKLLTVSGKNISLGPQNVFCDV   838
Chicken_PA      PQGCYLQLEFRYPLTPESLTVWVTFVSPDWDSSGAVNDVKLLTVSGKNISLGPQNVFCDI   876
Frog_PA         PHGCHLQLEFSHAVVPQSLTIWVTFVNTEWDTSGAVVDVKLLMVNGKELSLGPQNIFCDV   834
Zebraf_PAa      LQGCMLQLEFAYAVVPDSLTVWVTFSSQDETVLPAIHNIILLTVGGNNLSLGPSNLFCDT   833
Zebraf_PAb      PQGCMLQLEFAYPLLPDSLSVWVTFFSLEDTGLPAIQDILLLTLSGNNVSLGPQYVFCDT   829
                 :** *:***  :  :  *:**::****  .  :      *:  ::  **  :.*:::****.  :***

Human_PA        PLTIRLW--DVGEEVYGIQIYTLDEHLEIDAAMLTSTADTPLCLQCKPLKYKVVRDPPLQ   895
Mouse_PA        PLTIRLR--DVGEEVYGIQIYTLDEHLEIDAAMLTSTVDSPLCLQCKPLQYKVLRDPPLL   892
Dog_PA          PLTIKLR--DVGEEVSGIQIYTLDEHLEIDAAMLTSIADSPLCLECKPLQYKVVRDPPLH   896
Chicken_PA      PLTIKLDAGQVGEEVYGIQIYTLDEHLEIDAAMLSSVPHSTLCTDCKPIQYKVVRDPPFQ   936
Frog_PA         PLTIKLDNKDINEEVFGIQIYTKDNLMEIDAAMITSVPNSPLCAGCMPVHYKVLRDPPVE   894
Zebraf_PAa      PLTIKLD---VQEKVYGVQFYTMEQHLEIDATLLTSKPDCPLCGDCEPLHFRLLRQPPFS   890
Zebraf_PAb      LLTLKLD---VKEEVYGVQFFTMEQHLEIDATLLTSKPDNPLCRSCHPLRYRLLRQPPFT   886
                 **::*     : *:* *:*::* :: :****::::*   .   **   *  *::::::*:**.

                                                               >-M3 (S933-G995)
Human_PA        MDVA-S-ILHLNRKFVDMDLNLGSVYQYWVITISGTEESEPSPAVTYIHGSGYCGDGIIQ   953
Mouse_PA        EDVA-S-LLHLNRRFMDTDLKLGSVYQYRIITISGNEESEPSPAAIYTHGSGYCGDGVIQ   950
Dog_PA          VDVA-S-LLHLHRRFTDTDLSLGSTYQYWVITISGSEESEPSPAAIYIHGSGYCGDGIIQ   954
Chicken_PA      SGSP-VVISNLSRRFIDMELSDSTTYTYQVIVVSGAEESEPSPELVYISGSGYCGDGVIQ   995
Frog_PA         GEVP-SLISNLHRRYTDTNLKLNSIYKYQVVTMSGNKESEPSPELVYQHGSGYCGDGIIQ   953
Zebraf_PAa      NSKQGIMLSDSTRRYTDRDVEYGGIYTYQVQTVSSRSESEPSPPFTHRIGAPYCGDGFIQ   950
Zebraf_PAb      HSPHGIIIQEPTLRYEDRDLNPDETYTYHVLTVSRRAESEPSPPLVHQLGSPYCGDGLIQ   946
                        :  .     ::  *  ::.  .    *  *  :  .:*    ******     :   *:  *****.**

                                                 >-M4 (D996-G1018)
Human_PA        KDQGEQCDDMNKINGDGCSLFCRQEVSFNCIDEPSRCYFHDGDGVCEEFEQKTSIKDCGV   1013
Mouse_PA        KDQGEECDDMNKVNGDGCSLFCKQEVSFNCIDEPSRCYFHDGDGMCEEFEQKTSIKDCGV   1010
Dog_PA          KSQGEECDDMNKLNGDGCSLFCRQEDSFNCIDEPSRCYFHDGDGVCEEFEQKTSIKDCGV   1014
Chicken_PA      TDLGEECDDMNKINGDGCSLFCLQELSFNCIDEPSRCYFHDGDGVCEEFEQMTSIKDCGV   1055
Frog_PA         EDLGEECDDMNKLNGDGCSFFCRQEVSFNCLDEPSRCYFHDGDGVCEEFERMTSIKDCGV   1013
Zebraf_PAa      RSLGEECDDLNAMNGDGCSSHCKKEPFFNCIDEPSMCYYYDGDGVCEDFERETSVKDCGL   1010
Zebraf_PAb      KYLGEECDDMNFANGDGCSNQCKKEPLFNCVDQPSLCYFYDGDGVCEDFEREASVRDCGL   1006
                   **:***:*   ******   *  :*   ***:*:**  **::****:**:**:  :*::***:

                     >-M5 (F1019-F1181)
Human_PA        YTPQGFLDQWASNASVSH-QDQQCPGWVIIGQPAASQVCRTKVIDLSEGISQHAWYPCTI   1072
Mouse_PA        YTPQGFLDQWASNASVSH-QDQQCPGWVVIGQPAASQVCRTKVIDLSEGISQHAWYPCTI   1069
Dog_PA          YTPQGFLDQWASNASVSH-QDQQCPGWVIIGQPAASQVCRTKVIDLSEGISQHAWYPCTI   1073
Chicken_PA      YTPKGFLDQWASNVSVSHHSDQQCPGWVVIGQPAATQTCRTKVIDLNDGVSQYAWYPCTA   1115
Frog_PA         YTPKGFLDQWASNVSVSHQSEQLCPGWVVIGQPAATQVCLTKVLDLNDALSQYAWYPCTV   1073
Zebraf_PAa      YTPNGFLDQWATVVEVSHEERLYCPADVAAGYPAITKTCQSKVFDLSDGVSQYAWFPCRE   1070
Zebraf_PAb      YTPSGFLDQWATAVEVSNEEAIHCPAEVAAGYPAVTKSCQSKVFDLSHGVSQYAWFPCEE   1066
                ***.*******:  ..**:  .     **.  *   *  **  ::  *  :**:**...:**:**:**

Human_PA        SYPY-SQLAQTTFWLRAYFSQPMVAAAVIVHLVTDGTYYGDQKQETISVQLLDTKDQSHD   1131
Mouse_PA        TYPY-YHLPQTTFWLQTYFSQPMVAAAVIIHLVTDGTYYGDQKQETISVQLLDTKDQSHD   1128
Dog_PA          SYPYYSQMAQTTFWLRAYFSQPMVAAAVIVHLVTDGTYYGDQKQETISVQLFDTKDQSHD   1133
Chicken_PA      NFQY-SHMAQTIFWLKAYFSTPMVAAAVLVHLVTDGTYYLDQKQETIGVQLFDTKEQSHD   1174
Frog_PA         TTHF------VNFWLKAYFSRPVVVAAVIVHLVTDGTYINDQKPETISVQLIDTKEQLHD   1127
Zebraf_PAa      AH---SVPWYPPYWLKAYFSHPMVAAAVIIHLAADGTSYMDQTQCNITVQLIDTKEAIHS   1127
Zebraf_PAb      SQ---QQSW--TYWLKAYYAHPMVAAAVIIHLAADGTADMDQTQHNITVHLVDTKDQTHP   1121
                            :**::*::  *:*.***::**.:***    **.    .*  *:*.***:   *
```

```
                                                                    >-M6 (D1182-1214)
Human_PA        LGLHVLSCRNNPLIIPVVHDLSQPFYHSQAVRVSFSSPLVAISGVALRSFDNFDPVTLSS   1191
Mouse_PA        LGLHVLSCRNNPLIIPVVHDLSQPFYHSQAVHVSFSSPLVAISGVALRSFDNFDPVTLSS   1188
Dog_PA          LGLHVLSCRNNPLIIPVVHDLSQPFYHSRAVRVSFSSPLVAISGVALRSFDNFDPVTLSS   1193
Chicken_PA      LGVHVLSCRNNPLIIPVIHDLSHPFYHTQAVLISFSSQFVAISGVALRSFHNFDPITVSS   1234
Frog_PA         LGVHILSCRNNPLVISVMHDLSRPFYHSQTVLVSFTSNFVAISGVALRSFHDFDPVTISS   1187
Zebraf_PAa      LGDWRLSCRTNPLVIPVSHDLSMAFYHTKAILLMFRSEFVAISGVGLRSFQFFDPITISG   1187
Zebraf_PAb      LGDSRLSCRNNPLVIPVSHDLSVAFYRTKAIIVSFSSPLVAISGVGLRSFQYFDPITISG   1181
                **   ****.***:* * ****  **::::: : * * :******.****. ***:*:*.

                              >-SCR1 (C1215-V1283)
Human_PA        CQRGETYSPAEQSCVHFACEKTDCPELAVENASLNCSSSDRYHGAQCTVSCRTGYVLQIR   1251
Mouse_PA        CQRGETYSPAEQSCVHFACQAADCPELAVGNASLNCSSNHHYHGAQCTVSCQTGYVLQIQ   1248
Dog_PA          CQRGETYSSAEQSCVHFACEAADCPELAVENASLNCSSSDRYHGARCTVSCRTGYVLQIQ   1253
Chicken_PA      CQRGQTYSPAEQSCVHYSCEATDCQKLEIDNALLNCTGGGWYNGAQCNVSCRTGYILQVQ   1294
Frog_PA         CQRGEIYSAVEQSCVHYSCEATDCQELEIENSDVKCTS-GHYNGAQCEVTCHTGYILQIH   1246
Zebraf_PAa      CQSNEIYNPMGQSCVHYSCEAIDCQEPLIRNAEVECSDGGYFNGARCTVVCNCGYVLQIH   1247
Zebraf_PAb      CQSNEIYNPMGQSCVHYSCDAIECQKPMVSNAEMRCNSPGYFNGARCTITCNRGYTLKIH   1241
                ** .: *.   *****::*:  :* :  : *: :.*..   ::**:* : *. ** *:::

                                    >-SCR2 (D1284-L1344)
Human_PA        RDDELIKSQTGPSVTVTCTEGKWNKQVACEPVDCSIPDHHQVYAASFSCPEGTTFGSQCS   1311
Mouse_PA        RDDELIKSQVGPSITVTCTEGKWNKQVACEPVDCGIPDHHHVYAASFSCPEGTTFGRRCS   1308
Dog_PA          RDDELIKSQVGPSVTVTCSEGKWNKQVACEPVDCGIPDQHHVYAASFSCLKGTTFGSKCS   1313
Chicken_PA      RDDDLSKSQTESSITMTCTDGKWSKLVTCEPVDCGVPDQYHVYPATFNCSEGTTYGKKCS   1354
Frog_PA         RDDDLLKTQFESHIILTCRDGKWTKQVTCEPVDCGFPDKTHIHPATFSCPQGTTYGKQCT   1306
Zebraf_PAa      RDDDIIKSQTDSSVTITCADRKWNKQVSCEPVDCGLPDKYHVHPTIFDFPEGTTYGKKST   1307
Zebraf_PAb      RDDDIIKTQSESTVTLTCADGKWNKQVTCEPVDCGRPDKYHVHPAVFEFSEGTTYGKKCT   1301
                ***:: *:*    : :** : **.* *:******. **: ::: : *.  :***:* :.:

                                     >-SCR3 (M1345-V1413)
Human_PA        FQCRHPAQLKGNNSLLTCMEDGLWSFPEALCELMCLAPPPVPNADLQTARCRENKHKVGS   1371
Mouse_PA        FQCRHPAQLKGNNSFLTCMEDGLWSFPEALCELMCLAPPPVPNADLQTARCRENKHKVGS   1368
Dog_PA          FQCRHPAQLKGNNSLLTCMEDGLWSFPEALCELMCLAPPPVPNADLQTARCRENKHKVGS   1373
Chicken_PA      FTCRPPALLKGNNSNLTCMEDGLWSFPEALCELMCRAPSIVPNADLQTTRCLEDKHKVGS   1414
Frog_PA         FQCRPPAQLRGTNNMLTCLEDGLWSFPESICELMCLAPPSLPNAILQTARCLDDGHKVGS   1366
Zebraf_PAa      FQCKEPAQLVGFNNTLTCMEDGLWSFPEALCELRCPVPPPVPNAVLQTKRCNDTGLKVGT   1367
Zebraf_PAb      FQCREPAQLVGSNNVLTCMEDGMWSFPEALCELRCPIPPPVPNAVLQTKRCNATGLKVGS   1361
                * *: ** * * *. ***:***:*****::*** *  *  :*** *** **     ***:

                                                                 >-SCR4 (T1414-
1474)
Human_PA        FCKYKCKPGYHVPGSSRKS-------KKRAFKTQCTQDGSWQEGACVPVTCDPPPPKFHG   1424
Mouse_PA        FCKYKCKPGYHVPGSSRKS-------KKRAFKTQCTQDGSWQEGTCVPVTCDPPPPKFHG   1421
Dog_PA          FCKYKCKPGYHVPGSSRKS-------KKRAFKTQCTQDGSWQEGACVPVTCDPPPPKFHG   1426
Chicken_PA      FCKYKCKPGYHVPGSSRKA-------RKRAFKIQCTQDGTWLPGACVPVTCDPPPSKFHG   1467
Frog_PA         FCKYRCKQGFHVNDPLKKT-------KKKAFKTQCMEDGSWQLGKCVPITCEPPHPKFRG   1419
Zebraf_PAa      LCKYKCKPGYHVANKP----------KRRAFKRQCTEDGRWLEGSCEAVTCPAPPSVYYG   1417
Zebraf_PAb      FCKYKCKPGYHVPHIDKPKRSVPVMYHRRAFKRQCTEDGSWQFGECVAVTCEPPPPIFHG   1421
                :***:** *:**               :::*** ** :** *  * *  :**  *   : *

                                                                       >-SCR5
Human_PA        LYQCTNGFQFNSECRIKCEDSDAS--------QGLGSNVIHCRKDGTWNGSFHVCQEMQG   1476
Mouse_PA        LYQCTNGFQFNSECRIKCEDSDAS--------QGRGSNIIHCRKDGTWSGSFHVCREMQG   1473
Dog_PA          LYQCTNGFQFNSECRIKCEDSDAA--------QGRGSNIIHCRKDGTWSGSFHLCQEMQG   1478
Chicken_PA      LYQCSNGFQFNSECRIKCEDDDSQ--------SGRGSNVIHCRKDGTWSGSFHLCREMQG   1519
Frog_PA         LYQCTNGFQFNSECKLMCEESNSQ--------SDKVNNVIQCRKDGTWSGSFHLCQNIQG   1471
Zebraf_PAa      MYQCTDGFRFDSTCWIDCDGANHTLTQPPPCKQGAGSNVIRCRKDGNWTGSFRLCPQLKG   1477
Zebraf_PAb      TYKCTNGFQFNSDCWIDCNRSNHTA---------PSTNVIRCRKDGNWTGSFRLCPNLTG   1472
                 *:*::**:*:* * : *:  :              .*:*:*****.*.***::* :: *
```

```
                (Q1475-C1554)
Human_PA        QCSVPNELNSNLKLQCPDGYAIGSECATSCLDHNS-----ESIILPMNVTVRDI--PHWL   1529
Mouse_PA        QCSAPNQLNSNLKLQCPDGYAIGSECAISCLDHNS-----ESIILPVNLTVRDI--PHWM   1526
Dog_PA          QCSAPDQLNSNLKLQCPEGYAIGSECVTSCLDHNS-----ESIILPVNVTVRDI--PHWL   1531
Chicken_PA      QCALPTQLNSHLKLQCSGGYGIGAECTTSCLDHSH-----EPILLRVNETVQDI--QHWM   1572
Frog_PA         QCPPPYHMNNSMKIHCSEGYNIGAECMPICLNHNVEDNKMEAVILPANMTEKNL--PHWM   1529
Zebraf_PAa      QCSLPQNLHPSIRISCKKGHGIGEECELSCRDSNN-----DVVILPGNMTTSSIMRHHWM   1532
Zebraf_PAb      QCSLPQNLSPNIHLSCKDGHGIGKECEVSCRDASS-----SVVLLPSNMSVAAVMKDHWW   1527
                **  * .:   ::: *  *: ** **   * : .      . ::*  * :   :   **

                                          [1556-1568]
                                         >-C-term (V1555-G1627)
                                         >-LNR3 (V1555-C1583)--------<
Human_PA        NPTRVERVVCTAGLKWYPHPALIHCVKGCEPFMGDNYCDAINNRAFCNYDGGDCCTSTVK   1589
Mouse_PA        NPTRVQRIVCTAGLQWYPHPALIHCVKGCEPFMGDNYCDAINNRAFCNYDGGDCCTSTVK   1586
Dog_PA          NPTRVERVVCTAGLKWYPHPALIHCVKGCEPFMGDNYCDAINNRAFCNYDGGDCCASTVK   1591
Chicken_PA      NPQRVKSVVCTAGLKWYPHPSLIHCVKGCEPFMGDNYCDSINNRAFCNYDGGDCCASTVK   1632
Frog_PA         SPLRVKKIICTAGLQWYPLPGELYCIKSCEPFMGDNYCDGMNNRAFCNYDGGDCCVSTVK   1589
Zebraf_PAa      NPPKVKHIVCTMGLKWYPHPEVLHCIKGCEPFMGDNYCDAINNRAYCNYDGGDCCHSTVK   1592
Zebraf_PAb      NPPKVKNIVCTMGLKWYPNPEALHCIKGCEPFMGDNYCDAINNRAFCNYDGGDCCHSTVK   1587
                .* :*: ::** **:*** *  ::*:*.***********.:****:********* ****

                [1590-1599]
Human_PA        TKKVTPFPMSCDLQGDCACRDPQAQEHSRKDLRGYSHG    1627
Mouse_PA        TKKVTPFPMSCDLQNDCACRDPEAQEHNRKDLRGYSHG    1624
Dog_PA          TKKVTPFPMSCDLQGDCACRDPQAQEHSRKDLRGYSHG    1629
Chicken_PA      TKKVTPFPMSCDLQGECACRDPNAQEHNQKDLRGFSLG    1670
Frog_PA         TKMVTPFPANCDLQGECACLDPKAQENIHKDAHHPSLG    1627
Zebraf_PAa      TKKVIPFPMSCDIRRECACRDPNALENR-KDEHLHSLG    1629
Zebraf_PAb      TKKVIPFPMSCDLREDCACRDPDAQENN-KGARHRSIG    1624
                ** * *** .**:: :*** **.* *:  *. :  * *
```

b

```
                                                            >-start of mature STC2
Human_S2        ------------------MCAERLGQFMTLAL--VLATFDPARGTDATNPPEGPQDRS--   38
Mouse_S2        ------------------MCAERLGQFVTLAL--VFATLDPAQGTDSTNPPEGPQDRS--   38
Dog_S2          ------------------MCAERLGQFVTLAL--VLATFDLARGTDATNPPEGPQDRG--   38
Chicken_S2      ------------------MCAGLRGELPALALLLLLARAAAAAGTEATRPPEGQPERT--   40
Frog_S2         ------------------MCVELLNRLVTLGL--VFASFSAAFGTDATDLPENAPERVVP   40
Zebraf_S2a      ----------------------MLIKFTLS--LLLLSVLGEVVGTDNPDVHESHPEKP--   34
Zebraf_S2b      MLQWSGSPRQRYIQQRSSPSRDMRAQLT-LGVFILFLAVRQTHTTESASAHDTSQEKSL-   58
                                         .:       ::     .  *:     :   ::

                      [45-58]              [60-66]                     [86-107]
Human_S2        SQQKGRLSLQNTAEIQHCLVNAGDVGCGVFECFENNSCEIRGLHGICMTFLHNAGKFDAQ   98
Mouse_S2        SQQKGRLSLQNTAEIQHCLVNAGDVGCGVFECFENNSCEIQGLHGICMTFLHNAGKFDAQ   98
Dog_S2          SQQKGRLSLQNTAEIQHCLVNAGDVGCGVFECFENNSCEIRGLHGICMTFLHNAGKFDAQ   98
Chicken_S2      PQQKGRLSLQNTAEIQHCLVNAGDVGCGVFECFENNSCEIRGLHEICMTFLHNAGKFDAQ   100
Frog_S2         VQQKTRLSLQNSAEIQHCLVNAGDVGCGVFECFENNSCEIRGLHEICMTFLHNAGKFDAQ   100
Zebraf_S2a      ASQKGRLSLQNTAEIQHCLVSAGDVGCGVFECFENNSCEIRGLQEICMTFLHNAGKFDSQ   94
Zebraf_S2b      NIHKKRLSLQNTAEIQQCLVNAGDVGCGMFECFNNNSCEIRGLHDICMTFLHNAGKFDSQ   118
                  :* ******:****:***.*******:****:******:**: *************:*

                                   [118-124]
                                    C120
Human_S2        GKSFIKDALKCKAHALRHRFGCISRKCPAIREMVSQLQRECYLKHDLCAAAQENTRVIVE   158
Mouse_S2        GKSFIKDALRCKAHALRHKFGCISRKCPAIREMVFQLQRECYLKHDLCSAAQENVGVIVE   158
Dog_S2          GKSFIKDALKCKAHALRHRFGCISRKCPAIKEMVFQLQRECYLKHDLCSAAQENTRVMVE   158
Chicken_S2      GKSFIKDALKCKAHALRHKFSCISRKCPAIKEMVFQLQRECYLKHDLCSAAKENVQVIVE   160
Frog_S2         GKSFIKDALKCKAHALRHKFSCISRKCPAIKEMVYQLQRECYVKHDLCSAAKENVQVIVE   160
Zebraf_S2a      GKSFIKDALKCMAHGLRHKFSCISRKCLAIKDMVFQLQRECYMKHNLCSAAKDNVNVMVE   154
Zebraf_S2b      GKSFIKDTLKCMAHGLRHKFSCVSRKCVAVKEMVFQLQRECYLKHNLCLAVRENVNVMVE   178
                *******:*:* **.***:*.*:**** *:::** *******:**:** *.::*. *:**
```

```
Human_S2        MIHFKDLLLHEPYVDLVNLLLTCGEEVKEAITHSVQVQCEQNWGSLCSILSFCTSAIQKP   218
Mouse_S2        MIHFKDLLLHEPYVDLVNLLLTCGEDVKEAVTRSVQAQCEQSWGGLCSILSFCTSNIQRP   218
Dog_S2          MIHFKDLLLHEPYVDLVNLLLTCGEEVKEAITHSVQAQCEQNWGSLCSILSFCTSAIQRP   218
Chicken_S2      MIHFKDLLQHEPYVDLVNILLTCGEEVKKAITRSVQAQCEQNWGSLCSILSFCTSSMHGD   220
Frog_S2         MIHFKDLLLHEPYVDLVNLLLTCGEEVRDAITRSVQAQCAQNWGSLCYILSFCTSAMQGD   220
Zebraf_S2a      MIHFQDLFPKGPYVELVNILLGCGQEVKEAITRSVRLQCEQNWGALCDSLSFCTSMTAAP   214
Zebraf_S2b      MIHFKDLFPKGPHVELVNILLGCGEEVRVAIGRRIRTQCEQNWGALCGTLSLCALGKVEN   238
                ****:**: : *:*:***:** **::*: *: : :: ** *.**.**  **:*:

Human_S2        P---TAPPERQPQVDRTKLSRAHHGEAGHHLPEPS-S-RET--------------GRGAK   259
Mouse_S2        P---TAAPEHQPLADRAQLSRPHHRDTDHHLTA----------------------NRGAK   253
Dog_S2          P---TVPPERQLQVDRAKLSKTHHGEAGHHVSEPS-S-WET--------------GRGTK   259
Chicken_S2      A---VLSPEK----KTGEASKAAAGRGDVLVPAEPEH-RES--------------SRAAK   258
Frog_S2         S---AAMLSPSPPKKQGDSIKSFRSQSEPPSQGDAERDGAK--------------FSKND   263
Zebraf_S2a      A---SGGHERRPALTSHSDGEHHK----------------S--------------ARQGD   241
Zebraf_S2b      QASSTLAPVPDVTTTPGANTNALHPTSFPHPEADGQAVWTLTETREEENLSSGPQDNSSD   298
                                    .                                      .

Human_S2        GERGSKSHPNAHARGRVGGLGAQGPSGSSEWEDEQSEYSDIRR-----    302
Mouse_S2        GERGSKSHPNAHARGRTGGQSAQGPSGSSEWEDEQSEYSDIRR-----    296
Dog_S2          GEGGSKSHLNAHARGRAVGQGGQGTSGSSEWEEEPSEYSDIRR-----    302
Chicken_S2      GERGARGHLNAHTRVKAGAHGPKGAHGIMDRTDELSDFSDIRRSSCVL    306
Frog_S2         KDKNTKAHSNVHARAKTG-YSPKLAFSAMERADEPAGLSDIRR-----    305
Zebraf_S2a      KEKPGKAGFNTQMRIRSQGMRR-ASLDAVVAEQEDSKISDIRR-----    283
Zebraf_S2b      AQKELKRSPNATKRR---------------------------------    313
                 :   :   *.  *
```

Supplementary Fig. 18 Alignments of PAPP-A and STC2 sequences from multiple species. a Clustal Omega (1.2.4) multiple sequence alignment of preproPAPP-A from selected species (UniProt IDs: Human PAPP-A, Q13219; mouse PAPP-A, Q8R4K8; dog PAPP-A, A0A8I3NLM1; chicken PAPP-A, F1N850; xenopus PAPP-A, F7C2R5; zebrafish PAPP-Aa, M1XYM1; zebrafish PAPP-Ab, M1WXG8). Domain boundaries, C732, and selected motifs are indicated. **b** Clustal Omega (1.2.4) multiple sequence alignment of preSTC2 from the same species as in a (UniProt IDs: Human STC2, O76061; mouse STC2, O88452; dog STC2, A0A8I3MD84; chicken STC2, E1BRJ2; xenopus STC2, Q0V9Z1; zebrafish STC2a, A1L1M8; zebrafish STC2b, A0A0R4IS34). C120 is indicated. In both alignments, cysteine residues are shaded (grey). Regions involved in PAPP-A-STC2 interactions were identified by visual inspection of the structure and are indicated by highlighting and residue numbers in brackets above. PAPP-A and STC2 residues in regions with turquoise highlighting interact with each other, and PAPP-A and STC2 residues in regions with pink highlighting interact with each other.

Supplementary Table 1. Estimated resolution in regions of PAPP-A and STC2.*

Sequence region	Estimated resolution range (Å)	Primary map for subunit region	Additional maps for model building of dimer complex
**PAPP-A**			
81-324 (LamG)	3-4	MAP1	MAP2
325-410 (CD)	3-4	MAP1	MAP2
411-473 (LNR1-2)	3-5	MAP1	MAP2
474-671 (CD)	3-6	MAP1	MAP2
672-1014 (M1-M4)	3-4	MAP1	MAP2
1015-1281 (M5-M6, SCR1)	5-8	MAP3	MAP2
1282-1344 (SCR2)	3-4	MAP1	MAP2
1345-1554 (SCR3-5)	5-8	MAP2	MAP1
1555-1583 (LNR3)	4-5	MAP1	MAP2
1584-1617 (C-term)	4-7	MAP1	MAP2
**STC2**			
44-211	3-5	MAP1	MAP2

*Estimated resolution in regions of PAPP-A and STC2. The domains corresponding to the specified regions are given in parentheses. Resolution ranges are based on the local resolution maps of the indicated primary maps. Local resolution values were interpolated onto the atomic model and resolution ranges estimated by manual inspection of resolution in each region. Based on residue count, 32% of the full dimer complex was solved at 3.04 Å resolution (MAP1), 49% was solved at 4 Å resolution (MAP2), and 15% was solved at 5.1 resolution (MAP3). 4% of the structure remains unsolved.

Supplementary References

1. Overgaard MT*, et al.* Expression of recombinant human pregnancy-associated plasma protein-A and identification of the proform of eosinophil major basic protein as its physiological inhibitor. *J Biol Chem* **275**, 31128-31133 (2000).

2. Tunyasuvunakool K*, et al.* Highly accurate protein structure prediction for the human proteome. *Nature* **596**, 590-596 (2021).

3. Oxvig C, Sand O, Kristensen T, Kristensen L, Sottrup-Jensen L. Isolation and characterization of circulating complex between human pregnancy-associated plasma protein-A and proform of eosinophil major basic protein. *Biochim Biophys Acta* **1201**, 415-423 (1994).

4. Lang R*, et al.* Substrate specificity determinants of human macrophage elastase (MMP-12) based on the 1.1 A crystal structure. *J Mol Biol* **312**, 731-742 (2001).

5. Browner MF, Smith WW, Castelhano AL. Matrilysin-inhibitor complexes: common themes among metalloproteases. *Biochemistry* **34**, 6602-6610 (1995).

6. Maskos K*, et al.* Crystal structure of the catalytic domain of human tumor necrosis factor-alpha-converting enzyme. *Proc Natl Acad Sci U S A* **95**, 3408-3412 (1998).

7. Orth P*, et al.* Crystal structure of the catalytic domain of human ADAM33. *J Mol Biol* **335**, 129-137 (2004).

8. Tallant C, Garcia-Castellanos R, Seco J, Baumann U, Gomis-Ruth FX. Molecular analysis of ulilysin, the structural prototype of a new family of metzincin metalloproteases. *J Biol Chem* **281**, 17920-17928 (2006).

9. Guevara T, Rodriguez-Banqueri A, Ksiazek M, Potempa J, Gomis-Ruth FX. Structure-based mechanism of cysteine-switch latency and of catalysis by pappalysin-family metallopeptidases. *IUCrJ* **7**, 18-29 (2020).

10. Vardar D, North CL, Sanchez-Irizarry C, Aster JC, Blacklow SC. Nuclear magnetic resonance structure of a prototype Lin12-Notch repeat module from human Notch1. *Biochemistry* **42**, 7061-7067 (2003).

11. Laskowski RA, Hutchinson EG, Michie AD, Wallace AC, Jones ML, Thornton JM. PDBsum: a Web-based database of summaries and analyses of all PDB structures. *Trends Biochem Sci* **22**, 488-490 (1997).
