# Supplementary material for: Structure of the proteolytic enzyme PAPP-A with the endogenous inhibitor stanniocalcin-2 reveals its inhibitory mechanism
Source: Nat Commun. 2022 Oct 18;13:6084. doi: 10.1038/s41467-022-33698-8 (PMC9579167; doi:10.1038/s41467-022-33698-8)
Supplement: Supplementary file 3 — Description of Additional Supplementary Files/ Legends [file 41467_2022_33698_MOESM3_ESM.pdf]

Description of Additional Supplementary Files

File Name: Supplementary Data 1
Description: Peptides identified by mass spectrometry of a tryptic digest of PAPP-A·STC2 complex.

File Name: Supplementary Movie 1
Description: **Normal mode 2 of the 3D variability analysis of the PAPP-A·STC2 complex.** The 2-fold axis of the complex is in the center perpendicular to the viewed plane, with M6 in the foreground. The most pronounced variability is observed in the SCR region, but significant flexibility is also evident in other regions. The motion appears have a hinge located in the M6 domain.
