# Supplementary material for: Structure of the proteolytic enzyme PAPP-A with the endogenous inhibitor stanniocalcin-2 reveals its inhibitory mechanism
Source: Nat Commun. 2022 Oct 18;13:6084. doi: 10.1038/s41467-022-33698-8 (PMC9579167; doi:10.1038/s41467-022-33698-8)
Supplement: Supplementary file 6 — Reporting Summary [file 41467_2022_33698_MOESM6_ESM.pdf]

nature portfolio

Corresponding author(s): Claus Oxvig

Last updated by author(s): Sep 17, 2022

Reporting Summary

Nature Portfolio wishes to improve the reproducibility of the work that we publish. This form provides structure for consistency and transparency in reporting. For further information on Nature Portfolio policies, see our Editorial Policies and the Editorial Policy Checklist.

Statistics

For all statistical analyses, confirm that the following items are present in the figure legend, table legend, main text, or Methods section.

n/a	Confirmed	
☐	☒	The exact sample size (*n*) for each experimental group/condition, given as a discrete number and unit of measurement
☐	☒	A statement on whether measurements were taken from distinct samples or whether the same sample was measured repeatedly
☒	☐	The statistical test(s) used AND whether they are one- or two-sided<br>*Only common tests should be described solely by name; describe more complex techniques in the Methods section.*
☒	☐	A description of all covariates tested
☒	☐	A description of any assumptions or corrections, such as tests of normality and adjustment for multiple comparisons
☐	☒	A full description of the statistical parameters including central tendency (e.g. means) or other basic estimates (e.g. regression coefficient) AND variation (e.g. standard deviation) or associated estimates of uncertainty (e.g. confidence intervals)
☒	☐	For null hypothesis testing, the test statistic (e.g. *F*, *t*, *r*) with confidence intervals, effect sizes, degrees of freedom and *P* value noted<br>*Give P values as exact values whenever suitable.*
☒	☐	For Bayesian analysis, information on the choice of priors and Markov chain Monte Carlo settings
☒	☐	For hierarchical and complex designs, identification of the appropriate level for tests and full reporting of outcomes
☒	☐	Estimates of effect sizes (e.g. Cohen's *d*, Pearson's *r*), indicating how they were calculated

Our web collection on statistics for biologists contains articles on many of the points above.

Software and code

Policy information about availability of computer code

Data collection	EPU 2.11.1.11REL (commercial software from Thermo Fisher Scientific, www.thermofisher.com) was used for cryo-EM data collection.
Data analysis	cryoSPARC 3.3.2 (free licensed software from Structura Biotechnology, www.cryosparc.com), PHENIX 1.20.1 (free software (for academia) from the Phenix team - www.phenix-online.org), Coot 0.9.8.3 (free software (for academia) available from https://www2.mrc-lmb.cam.ac.uk/personal/pemsley/coot/), UCSF ChimeraX 1.3 (free software (for academia) available from https://www.cgl.ucsf.edu/chimerax), PyMOL v2.5.2 (https://www.pymol.org).

For manuscripts utilizing custom algorithms or software that are central to the research but not yet described in published literature, software must be made available to editors and reviewers. We strongly encourage code deposition in a community repository (e.g. GitHub). See the Nature Portfolio guidelines for submitting code & software for further information.

Data

Policy information about availability of data

All manuscripts must include a data availability statement. This statement should provide the following information, where applicable:

- Accession codes, unique identifiers, or web links for publicly available datasets
- A description of any restrictions on data availability
- For clinical datasets or third party data, please ensure that the statement adheres to our policy

The cryo-EM 3D maps haves been deposited in the Electron Microscopy Data Bank (EMDB) with the following accession codes: EMD-15220 [https://www.ebi.ac.uk/

pdbe/entry/emdb/EMD-15220] (MAP1), EMD-15217 [https://www.ebi.ac.uk/pdbe/entry/emdb/EMD-15217] (MAP2), EMD-15219 [https://www.ebi.ac.uk/pdbe/entry/emdb/EMD-15219] (MAP3), and EMD-15221 [https://www.ebi.ac.uk/pdbe/entry/emdb/EMD-15221] (composite map). The corresponding atomic models have been deposited in the Protein Data Bank (PDB) with the following accession codes: 8A7D [https://www.rcsb.org/structure/8A7D] (partial dimer model,) and 8A7E [https://www.rcsb.org/structure/8A7E] (full heterotetrameric PAPP-A·STC2 complex). Source data are provided with this paper.

Human research participants

Policy information about studies involving human research participants and Sex and Gender in Research.

Reporting on sex and gender	n/a
Population characteristics	n/a
Recruitment	n/a
Ethics oversight	n/a

Note that full information on the approval of the study protocol must also be provided in the manuscript.

Field-specific reporting

Please select the one below that is the best fit for your research. If you are not sure, read the appropriate sections before making your selection.

☒ Life sciences ☐ Behavioural & social sciences ☐ Ecological, evolutionary & environmental sciences

For a reference copy of the document with all sections, see nature.com/documents/nr-reporting-summary-flat.pdf

Life sciences study design

All studies must disclose on these points even when the disclosure is negative.

Sample size	No methods were used that would necessitate a predetermined sample size. Three biologically independent samples were used for each analysis.
Data exclusions	No data were excluded from the study.
Replication	Experiments were repeated three times (n = 3) or more as specifically specified in the text.
Randomization	Randomization was not used. All results from experiments were analyzed.
Blinding	Investigators were not blinded in the study as there is no bias for the data included in the study.

Reporting for specific materials, systems and methods

We require information from authors about some types of materials, experimental systems and methods used in many studies. Here, indicate whether each material, system or method listed is relevant to your study. If you are not sure if a list item applies to your research, read the appropriate section before selecting a response.

Materials & experimental systems

n/a	Involved in the study	
☐	☒	Antibodies
☐	☒	Eukaryotic cell lines
☒	☐	Palaeontology and archaeology
☒	☐	Animals and other organisms
☒	☐	Clinical data
☒	☐	Dual use research of concern

Methods

n/a	Involved in the study	
☒	☐	ChIP-seq
☒	☐	Flow cytometry
☒	☐	MRI-based neuroimaging

Antibodies

Antibodies used	The following murine monoclonal antibodies were used in the study:

Antibodies used

PAPP-A mAb 234-5
PAPP-A mAb PA6
PAPP-A mAb PA141
PAPP-A mAb PA1A
STC2 mAb STC221

The following rabbit polyclonal antibodies were used in the study:
Polyclonal rabbit(anti-PAPP-A)

All are in-house antibodies. The antibodies were produced by culture of the corresponding hybridoma clones followed by purification and determination of concentration, as detailed in Methods. The concentrations of the antibodies in the applications are specified in Methods. Polyclonal antibodies were produced by immunization of rabbits with purified human PAPP-A (Oxvig et al. 1994), and used as an antiserum at the dilution specified.

Validation

Primary antibodies were previously validated for specificity and application:

PAPP-A mAb 234-5 (immobilization of PAPP-A or SPR analysis), Mikkelsen et al. (ref. 67)
PAPP-A mAb PA6 (immunoassay), Botkjaer et al. (ref. 69)
PAPP-A mAb PA141 (detecting antibody in SPR analysis, immunoassay), Mikkelsen et al. (ref. 36)
PAPP-A mAb PA1A (control antibody for PAPP-A in SPR analysis), Gyrup et al. (ref. 68)
STC2 mAb STC221 (immunoassay), Jepsen et al. (ref. 21)
Rabbit anti(PAPP-A) (PAPP-A Western blotting), Jepsen et al. (ref. 70)

Eukaryotic cell lines

Policy information about cell lines and Sex and Gender in Research

Cell line source(s)	Cell line used: human embryonic kidney 293T cells (293tsA1609neo, ATCC).
Authentication	No authentication procedure for the cell line used.
Mycoplasma contamination	All cell lines used in the study were tested negative for mycoplasma infection.
Commonly misidentified lines (See ICLAC register)	No commonly misidentified cell lines were used in the study.
